# Investigation of yield, phytochemical composition, and photosynthetic pigments in different mint ecotypes under salinity stress

**DOI:** 10.1002/fsn3.2219

**Published:** 2021-03-15

**Authors:** Seyyed Jaber Hosseini, Zeinolabedin Tahmasebi‐Sarvestani, Hemmatollah Pirdashti, Seyed Ali Mohammad Modarres‐Sanavy, Ali Mokhtassi‐Bidgoli, Saeid Hazrati, Silvana Nicola

**Affiliations:** ^1^ Department of Agronomy Tarbiat Modares University Tehran Iran; ^2^ Sari Agricultural Sciences and Natural Resources University Sari Iran; ^3^ Department of Agronomy Faculty of Agriculture Azarbaijan Shahid Madani University Tabriz Iran; ^4^ Department of Agricultural, Forest and Food Sciences VEGMAP University of Turin Grugliasco Italy

**Keywords:** antioxidant activity, cluster analysis, essential oil, mint, photosynthesis, salinity stress

## Abstract

Salinity stress is one of the main limiting factors of medicinal plant growth and may affect their characteristics and chemical composition. In order to evaluate the response of different species of Iranian mint to salinity stress, an experiment was designed in greenhouse conditions. In this experiment, six Iranian mint species were cultivated in pots under different salinity stress including 0, 2.5, 5, and 7.5 dS/m. The chlorophyll indices (a, b, total, and a/b ratio), carotenoids, total anthocyanin, total phenolic and flavonoid content, antioxidant activity, dry matter yield, and essential oil content were measured in two different harvest stages. Salinity stress affected various measured traits. The results showed that despite the negative effect of salinity stress on photosynthetic pigments, in some ecotypes and species, photosynthetic pigments were not affected by salinity stress. The amount of total phenolic content, total flavonoid content, and total anthocyanin increased in response to salinity stress. The dry matter decreased under salinity stress, but the content of essential oil increased as a result of salinity stress increment. The results of PCA biplot showed that the E16 and E18 ecotypes were separated by a large distance. Among the various ecotypes, E18 had the most desirable traits which can be recognized as a salt‐tolerant ecotype. Also, *piperita* species was the best among the species in all salinity stress levels.

## Graphical abstract

Among the various ecotypes, E18 accounted for the most desirable traits in most of the studied traits and was recognized as a salt‐tolerant ecotype.

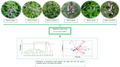

## 1 INTRODUCTION

Soil and water salinity is one of the most important environmental factors limiting the growth and yield of plant crops in the world, especially in arid and semiarid regions (Foster et al., 2018). Currently, millions of hectares of agricultural land in the world are unusable due to then increased salinity (Dagar & Minhas, 2016). Lack of proper management in irrigation, improper drainage, rising groundwater levels, use of salt water for irrigation, overuse of chemical fertilizers, and high evaporation rate are effective in development of saline soils and salinizing groundwater (Wu et al., 2014). Various environmental stresses, such as salinity, drought, temperature, and high light intensity, lead to the production of oxygen‐free radicals (Arora et al., 2016; Miller et al., 2018). Salinity stress leads to a number of morphological, physiological, biochemical, and molecular changes that have negative consequences in plant growth and productivity (Rivero et al., 2014). The plant responds differently to salinity stress which depends on the degree of ionic toxicity, changes in osmotic potential, stress duration, and plant species type (Arora et al., 2020; Taïbi et al., 2016). The salinity stress damage in plants is mainly caused by osmotic stress and leads to a decrease in cell water content, ionic toxicity, and impaired absorption of nutrients (Khalvandi et al., 2019). Photosynthesis is important in the production of secondary metabolites and dry matter. Growing in saline conditions reduces the photosynthetic activity in plants (Mahlooji et al., 2018). Chlorophyll pigments have a key role in photosynthesis. Chlorophyll content is considered as an effective indicator to monitor photosynthesis activity in plants (Taïbi et al., 2016). Therefore, by assessing the content of photosynthetic pigments under different salinity conditions, the effect of salinity stress on photosynthetic activity can be partially understood. In addition, anthocyanins are the nonphotosynthetic pigments in plants which can play a positive role against environmental stresses (Xu et al., 2017). The total phenolic content, total flavonoid content, and essential oils are also influenced by the salinity stress. Several studies showed that increasing the salinity stress leads to an increase in the content of total phenolic content, flavonoids, and essential oil content (Bistgani et al., 2019; Khalvandi et al., 2019; Vafadar Shoshtari et al., 2017). Furthermore, under salinity stress condition, the synthesis of secondary metabolites may be increased (Cui et al., 2019). In general, increasing salinity in the root environment reduces growth rate and yield. In the whole life cycle of a plant (from the beginning of growth to the production of maximum biomass), salinity affects all major processes including growth, photosynthesis, protein synthesis, and lipid metabolism (Negrão et al., 2017; Nxele et al., 2017). In stressful conditions, plants need to maintain turgor and water absorption for growth. This requires an increase in osmotic activity, which is achieved by adsorption from the soil or by the synthesis of metabolic solutions. This process also requires energy consumption, which may negatively influence plant growth (Isayenkov & Maathuis, 2019). High levels of sodium in the soil may impaired the plant's capacity to absorb potassium (Ahanger & Agarwal, 2017). Investigating the effects of various environmental conditions on the quantitative and qualitative performance medicinal plants has always been one the major research areas. The ultimate goal of cultivating medicinal plants is the production of secondary metabolites, and obviously the higher content of these metabolites, the more valuable the production process (Ahanger & Agarwal, 2017; Khalvandi et al., 2019). Previous studies have shown that one of the most important factors influencing the content of secondary metabolites in plants is the environmental stresses employed in their cultivation process (Banerjee & Roychoudhury, 2017). The protective role among the stress is the most important functions of secondary metabolites in plants (Khalvandi et al., 2019). These compounds help plants to overcome biotic stressors (e.g., pests and pathogens) and abiotic stress conditions such as salinity (Bennett & Wallsgrove, 1994; Cui et al., 2019). Different species of mint (Lamiacea family) are native to temperate regions, especially Europe, North America, and North Africa, which are widely cultivated around the world (Singh et al., 2015). Mint is among the most important medicinal plants widely cultivated and consumed on a global scale, mainly due to its high content of essential oils (Lawrence, 2006). This plant is used as a rich source of essential oils that has a broad nutritional application. In traditional medicine, it is conventionally used as an antiflatulent agent and food digestion aid (Lis‐Balchin & Hart, 1999; Sharathchandra et al., 1995). Also, mint has always been considered for its antiseizure, stimulant, invigorating, stomach upset, pain, and stomach ulcer properties, as well as its numerous health and nutritional values (Asghari et al., 2018; Chiou et al., 2020; Zimmerman & Yarnell, 2018). Khalvandi et al., (2019) showed that salinity stress increased the production of mint secondary metabolites. Also, they showed that antioxidant activity, total phenolic content, and total flavonoid content were affected by the salinity stress. They found that the amount of these metabolites increased with intensification of salinity stress. Other studies also showed that the chlorophyll a and b contents were reduced after the salinity stress in mint (Chrysargyris et al., 2019; Mohammadi et al., 2020; Tab atabaie & Nazari, 2007). Furthermore, it has been found in several studies that the content of essential oils in mint increased with the increasing salinity stress (Khalvandi et al., 2019; Vafadar Shoshtari et al., 2017; Tab atabaie & Nazari, 2007). Previous studies confirmed that the synthesis of dry matter in plants decreased in response to the salinity stress (Aziz et al., 2008; Oueslati et al., 2010; Wu et al., 2016). Genetic differences between different ecotypes and species make it possible to use this potential to select tolerant and sensitive ecotypes for specific conditions such as plant exposure to the salinity stress (Dwivedi et al., 2016). Different plant species and ecotypes have different properties, and this issue can be effectively used in plant breeding programs (Grover, 2012). Several studies focused on different plant species and ecotypes under salinity stress (Aziz et al., 2008; Wu et al., 2016).

Due to the importance of mint in different industries, also large‐scale cultivation in different climatic conditions and the increase in salinity of water/soil on the global scale, the main aim of this experiment was to investigate the effect of the salinity stress on the content of major phytochemicals and dry matter of different mint ecotypes.

## 2 MATERIALS AND METHODS

**FIGURE 1:**
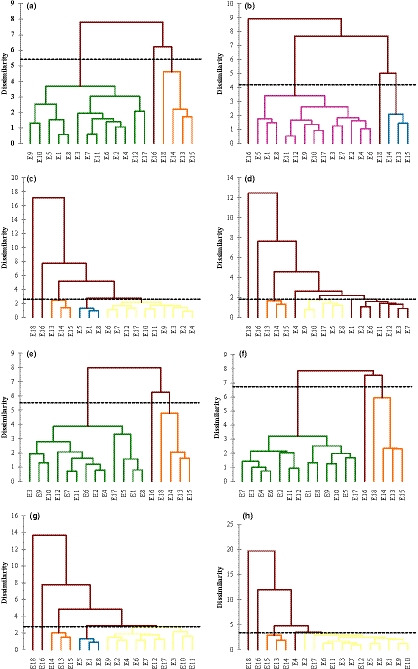
Dendrogram of hierarchical cluster analysis for 18 mint ecotypes. The hierarchical clustering was performed by XLSTAT software. UPGMA (unweighted pair group method with arithmetic mean) method was applied, and Euclidean distance was selected as the measurement. Dendrogram resulted from the all studied traits in 18 mint ecotypes. (A), control at first harvest; (B), 2.5 dS/m at first harvest; (C), 5 dS/m first harvest; (D), 7.5 dS/m first harvest; (E), control at second harvest; (F), 2.5 dS/m at second harvest; (G), 5 dS/m second harvest; (H), 7.5 dS/m second harvest

The ecotypes were provided from the Gene Bank of Research Institute of Forests and Rangelands. The seeds were sown in plastic pots (25 cm diameter and 30 cm height) filled with cocopeat (40%) and perlite (60%) and placed in a glasshouse with a temperature between 25 and 28°C, 16/8 day/night photoperiod and relative humidity of 60% throughout the experiment. The treatments included four levels of salinity (0, 2.5, 5, and 7.5 dS m^−1^), two harvesting time points, and 18 ecotypes (Table 1 and Figure 1). The experiment arranged a split factorial experimental design based on using randomized complete block design with four replications. The plants were irrigated with Hoagland nutrient solution (Hoagland & Arnon, 1950). The soil salinity stress was imposed 15 days after seed sowing. To prevent salinity shock to the seedlings, high salinity levels (5 and 7.5 dS m^−1^) were gradually applied (Reich et al., 2017). This was achieved in a way that the electrical conductivity of the solution increased stepwise by 2.5 dS m^−1^ to reach 5 and 7.5 dS m^−1^. Moreover, to prevent the accumulation of solutes in the culture medium, washing was carried out in certain periods. Eventually, first harvest took place 70 days after planting while the second harvesting took place when the plants entered into the flowering phase (67 days after the first harvest). Harvesting was conducted early in the morning to avoid the hottest hours of the day. The plants were weighted and then placed in an oven at 70°C for 48 hr to determine dry weight using a digital scale.

**TABLE 1:** Ecotype number, species, latitude, longitude, altitude, and place related to 18 mint ecotypes

Ecotype number	Species	Place	Latitude (*N*)	Longitude (E)	Altitude (m)
E_1_	*longifolia*	Mazandaran‐Hezar Jarib	36◦4,524''	53◦2,806''	389
E_2_	*longifolia*	Fars‐Shiraz	29◦7,447''	52◦4,590''	1,491
E_3_	*longifolia*	Kordestan‐Sanandaj, Dolatabad Village	35◦2000''	47◦9,999''	2039
E_4_	*longifolia*	Markazi‐Arak	34◦1,115''	49◦3,111''	2030
E_5_	*longifolia*	Golestan‐Ramyan	36◦5,513''	55◦0,655''	780
E_6_	*longifolia*	Tehran	35◦5,229''	52◦9,383''	1940
E_7_	*longifolia*	Zanjan	36◦4,235''	48◦0,703''	1,800
E_8_	*longifolia*	Semnan‐Damghan	36◦2,748''	54◦1738''	2,300
E_9_	*longifolia*	Ilam‐Dehloran	32◦5,307''	47◦0,030''	807
E_10_	*pulegium*	Markazi‐Tafresh	34◦4,703''	49◦5,800''	1,550
E_11_	*pulegium*	Markazi‐Khomein	33◦3,635''	50◦0,150''	1808
E_12_	*pulegium*	South Khorasan‐Sarbisheh	32◦3,223''	65◦1,139''	1817
E_13_	*pulegium*	Mazandaran	32◦3,703''	51◦2,590''	1662
E_14_	*spicata*	Isfahan‐Najafabad	32◦1854''	51◦4,307''	1652
E_15_	*spicata*	Yazd	31◦1588''	54◦3,089''	1,243
E_16_	*rotundifolia*	Ilam‐Ivan	33◦5,108''	46◦1,104''	1,142
E_17_	*mozafariani*	Hormozgan‐Bandar Abbas	27◦5,014''	56◦1805''	1,117
E_18_	*piperita*	Mazandaran‐Sari	36◦7,700''	53◦0,599''	1,255

### 2.1 Essential oil extraction

To extract and measure essential oils content, 50 g dried samples were subjected to hydro‐distillation method for 150 min using an all‐glass Clevenger‐type apparatus using the method proposed by British Pharmacopoeia (1988). When the samples were boiled, it was maintained in the minimum temperature required for boiling, and then, the collected extract (essential oils and distilled water) was dried under sodium sulfate to calculate essential oil content. The samples were kept at darkness and temperature of 4°C until analysis.

### 2.2 Measuring the content of total phenolic compounds

Gallic acid was used as the standard for measuring the content of total phenolic content compounds. A mixture of 100 μl of the extract (2 mg/ml) was mixed with 500 μl of Folin–Ciocalteu (10% by volume/volume). After 5 min, 500 ml of 7% sodium carbonate was added to the mixture and the absorption of the samples (after 2 hr in the dark) was measured at 765 nm by spectrophotometer (Analytik Jena, Spekol 1,300, Germany) (Haghighi et al., 2012).

### 2.3 Measuring the content of total flavonoid compounds

Quercetin was used as the standard for measuring the content of total flavonoid compounds. A mixture of 100 μl of the extract (2 mg/ml) was mixed with 50 μl of aluminum chloride (2% by volume/volume) and 100 μl of ammonium acetate (1 mol). After 10 min, the absorption of the samples was measured at 426 nm by spectrophotometer (Analytik Jena, Spekol 1,300, Germany) (Haghighi et al., 2012).

### 2.4 Determining antioxidant activity

DPPH (2,2‐Diphenyl‐1‐Picrylhydrazyl) and the standard substance ascorbic acid were used to evaluate the antioxidant properties of the extracts. First, 2 mg of the extracts was dissolved in 1 ml of methanol and then different concentrations (20, 40, 60, 80, 100 μg/ml) were prepared. 550 μl of DPPH solution was added to 50 μl of each of the prepared solutions and placed in the dark for 30 min. The absorption of all solutions and control samples was then measured using a spectrophotometer (Analytik Jena, Spekol 1,300, Germany) at 517 nm (Hazrati et al., 2019). Radical scavenging activity was evaluated based on the following equation:

$$\text{Percentage of radical scavenging activity}=\left(\text{Abs control}‐\text{Abs sample}\right)/\text{Abs control}\times 100$$

### 2.5 Measurement of chlorophyll and carotenoids content

Lichtenthaler's (1987) method was applied to measure chlorophyll and carotenoids content. To extract these pigments, 0.2 g of leaf samples was immersed in test tubes containing 80% acetone. The samples were filtered through filter paper and finally their absorption was read at 646.8, 663.2 and 470 nm by spectrophotometer (Analytik Jena, Spekol 1,300, Germany).

### 2.6 Total Anthocyanin measurement

Anthocyanins were extracted and measured based on Krizek et al. (1993) method. 0.2 g of leaf samples was homogenized in 3 ml 1% HCl–methanol solvent (1:99, v: v). The extract was then centrifuged, and the top solution was kept in the dark for one hour. The absorption was read at 550 nm by spectrophotometer (Analytik Jena, Spekol 1,300, Germany).

### 2.7 Statistical analysis

SAS 9.1 was used to analyze the data. The least significant differences (LSD) test was applied to compare the mean data. The cluster and principal component analysis were done using XLSTAT.

## 3 RESULTS AND DISCUSSION

### 3.1 Photosynthetic pigments

The results of the present experiment showed that the amount of chlorophyll a, b and total chlorophyll in different species was affected by salinity stress and decreased with increasing salinity stress (Table 2). The highest amount of chlorophyll a in all salinity stress levels was observed in *piperita*, *rotundifolia,* and *spicata* species, while the other species had the lowest amount of chlorophyll a. The results of the comparison between the first and second harvest stages showed that at salinity of 7.5 dS/m, the amount of chlorophyll a was higher in the second harvest. At control and 2.5 dS/m salinity stress levels, *piperita* and *spicata* showed the highest content of chlorophyll b and total chlorophyll, but with increasing salinity stress to 5 and 7.5 dS/m, only *piperita* species had the highest chlorophyll b and total chlorophyll content (Tables 3 and 4). Comparison between harvest stages also revealed that the chlorophyll b and total chlorophyll contents were higher at salinity of 7.5 dS/m in the second harvest. The highest chlorophyll a/b ratio was observed in *rotundifolia* species in all salinity stress levels, while other species had the lowest chlorophyll a/b ratio (Table 5).

**TABLE 2:** Content of chlorophyll a (µg/mL) related to different mint ecotypes and salinity stress levels at first and second harvest

Ecotypes	First harvest				Second harvest			
	Salt stress (dS/m)				Salt stress (dS/m)			
	0	2.5	5	7.5	0	2.5	5	7.5
E_1_	8.36 (±0.58) ^cd A^	7.94 (±0.58) ^c AB^	6.36 (±0.58) ^a‐d BC^	4.94 (±0.58) ^d C^	8.41 (±0.58) ^b‐d A^	8.01 (±0.58) ^c A^	6.66 (±0.58) ^a‐e AB^	5.16 (±0.58) ^e B^
E_2_	6.32 (±0.62) ^f A^	5.88 (±0.17) ^f A^	5.11 (±0.41) ^d A^	5.11 (±0.12) ^d A^	6.42 (±0.11) ^e A^	5.89 (±0.10) ^g AB^	5.27 (±0.52) ^e B^	5.35 (±0.12) ^de B^
E_3_	6.87 (±0.12) ^ef A^	6.25 (±0.22) ^ef AB^	5.38 (±0.44) ^d B^	5.38 (±0.22) ^cd B^	6.99 (±0.40) ^c‐e A^	6.38 (±0.15) ^e‐g A^	5.70 (±0.59) ^de A^	5.69 (±0.30) ^c‐e A^
E_4_	6.02 (±0.63) ^f A^	5.66 (±0.13) ^f A^	5.00 (±0.43) ^d A^	5.00 (±0.09) ^d A^	6.32 (±0.55) ^e A^	5.90 (±0.24) ^g A^	5.03 (±0.46) ^e A^	5.13 (±0.12) ^e A^
E_5_	7.89 (±0.23) ^de A^	7.50 (±0.30) ^cd A^	6.17 (±0.50) ^b‐d B^	6.17 (±0.43) ^bc B^	7.93 (±0.14) ^c‐e A^	7.51 (±0.13) ^cd A^	6.35 (±0.48) ^c‐e B^	6.45 (±0.15) ^cd B^
E_6_	6.03 (±0.56) ^f A^	5.79 (±0.23) ^f A^	5.10 (±0.53) ^d A^	5.10 (±0.18) ^d A^	6.16 (±0.60) ^e A^	6.05 (±0.14) ^fg A^	5.11 (±0.53) ^e A^	5.31 (±0.37) ^e A^
E_7_	6.47 (±0.11) ^ef A^	6.28 (±0.22) ^ef AB^	5.39 (±0.47) ^d B^	5.39 (±0.19) ^cd B^	6.71 (±0.50) ^de A^	6.43 (±0.30) ^e‐g A^	5.47 (±0.54) ^e A^	5.59 (±0.26) ^c‐e A^
E_8_	8.54 (±0.20) ^cd A^	7.94 (±0.18) ^c A^	6.37 (±0.59) ^a‐d B^	6.37 (±0.29) ^b B^	8.76 (±0.20) ^bc A^	8.08 (±0.33) ^c A^	6.41 (±0.44) ^b‐e B^	6.61 (±0.46) ^bc B^
E_9_	7.36 (±0.21) ^d‐f A^	6.77 (±0.20) ^de A^	5.76 (±0.43) ^cd B^	5.76 (±0.10) ^b‐d B^	7.39 (±0.55) ^c‐e A^	6.78 (±0.20) ^d‐g AB^	5.79 (±0.57) ^de B^	5.99 (±0.35) ^c‐e AB^
E_10_	7.25 (±0.29) ^d‐f A^	6.81 (±0.12) ^de A^	5.69 (±0.39) ^cd B^	5.69 (±0.23) ^b‐d B^	7.58 (±0.70) ^c‐e A^	7.00 (±0.20) ^de AB^	5.93 (±0.41) ^de B^	5.89 (±0.37) ^c‐e B^
E_11_	6.47 (±0.64) ^ef A^	6.28 (±0.11) ^ef A^	5.35 (±0.43) ^d A^	5.35 (±0.34) ^cd A^	6.58 (±0.53) ^de A^	6.33 (±0.15) ^e‐g A^	5.43 (±0.38) ^e A^	5.50 (±0.19) ^c‐e A^
E_12_	6.58 (±0.57) ^ef A^	6.25 (±0.14) ^ef A^	5.28 (±0.49) ^d A^	5.28 (±0.21) ^cd A^	6.64 (±0.19) ^de A^	6.36 (±0.15) ^e‐g AB^	5.43 (±0.47) ^e B^	5.55 (±0.38) ^c‐e AB^
E_13_	10.56 (±0.18) ^ab A^	10.45 (±0.42) ^a A^	7.99 (±0.51) ^a B^	7.99 (±0.14) ^a B^	10.74 (±0.37) ^a A^	10.67 (±0.31) ^a A^	8.08 (±0.51) ^ab B^	8.13 (±0.38) ^a B^
E_14_	9.65 (±0.61) ^bc A^	9.36 (±0.16) ^b A^	7.32 (±0.68) ^a‐c B^	7.32 (±0.47) ^a B^	9.89 (±0.63) ^ab A^	9.67 (±0.28) ^b A^	7.35 (±0.51) ^a‐d B^	7.53 (±0.13) ^ab B^
E_15_	10.74 (±0.43) ^ab A^	10.10 (±0.23) ^ab A^	7.63 (±0.62) ^ab B^	7.63 (±0.40) ^a B^	10.80 (±0.81) ^a A^	10.25 (±0.35) ^ab A^	7.64 (±0.57) ^a‐c B^	7.91 (±0.41) ^a B^
E_16_	10.56 (±0.73) ^ab A^	10.14 (±0.29) ^ab A^	7.57 (±0.79) ^ab B^	7.57 (±0.13) ^a B^	10.81 (±1.12) ^a A^	10.28 (±0.42) ^ab A^	7.69 (±0.58) ^a‐c B^	7.67 (±0.40) ^a B^
E_17_	7.36 (±0.13) ^d‐f A^	6.77 (±0.23) ^de A^	5.55 (±0.45) ^d B^	5.55 (±0.13) ^b‐d B^	7.60 (±0.26) ^c‐e A^	6.91 (±0.24) ^d‐f AB^	5.87 (±0.54) ^de BC^	5.69 (±0.13) ^c‐e C^
E_18_	11.23 (±0.32) ^a A^	10.78 (±0.37) ^a A^	8.00 (±0.83) ^a B^	8.00 (±0.14) ^a B^	11.50 (±0.66) ^a A^	10.89 (±0.19) ^a A^	8.28 (±0.86) ^a B^	8.08 (±0.42) ^a B^
Species								
*longifolia*	7.10 (±0.20) ^b A^	6.67 (±0.17) ^b A^	5.63 (±0.17) ^b B^	5.47 (±0.12) ^b B^	7.23 (±0.20) ^b A^	6.78 (±0.16) ^b A^	5.76 (±0.18) ^c B^	5.70 (±0.13) ^b B^
*pulegium*	7.72 (±0.48) ^b A^	7.45 (±0.46) ^b A^	6.08 (±0.35) ^b B^	6.08 (±0.31) ^b B^	7.88 (±0.49) ^b A^	7.59 (±0.47) ^b A^	6.22 (±0.35) ^bc B^	6.27 (±0.32) ^b B^
*spicata*	10.19 (±0.40) ^a A^	9.73 (±0.19) ^a A^	7.48 (±0.43) ^a B^	7.48 (±0.29) ^a B^	10.35 (±0.50) ^a A^	9.96 (±0.24) ^a A^	7.50 (±0.36) ^ab B^	7.72 (±0.21) ^a B^
*rotundifolia*	10.56 (±0.73) ^a A^	10.14 (±0.29) ^a A^	7.57 (±0.79) ^a B^	7.57 (±0.13) ^a B^	10.81 (±1.12) ^a A^	10.28 (±0.42) ^a A^	7.69 (±0.58) ^a B^	7.67 (±0.40) ^a B^
*mozafariani*	7.36 (±0.13) ^b A^	6.77 (±0.23) ^b A^	5.55 (±0.45) ^b B^	5.55 (±0.13) ^b B^	7.60 (±0.26) ^b A^	6.91 (±0.24) ^b A^	5.87 (±0.54) ^c B^	5.69 (±0.13) ^b B^
*piperita*	11.23 (±0.32) ^a A^	10.78 (±0.37) ^a A^	8.00 (±0.83) ^a B^	8.00 (±0.14) ^a B^	11.50 (±0.66) ^a A^	10.89 (±0.19) ^a A^	8.28 (±0.86) ^a B^	8.08 (±0.42) ^a B^
**Total mean**	**8.01 (±0.23) ^A^**	**7.61 (±0.21) ^A^**	**6.17 (±0.17) ^A^**	**6.09 (±0.14) ^B^**	**8.18 (±0.24) ^A^**	**7.74 (±0.21) ^A^**	**6.31 (±0.17) ^A^**	**6.29 (±0.14) ^A^**

Note

Results are represented as mean ± standard error. Means in a column and row followed by the different superscripts are significantly different at *p* ≤ .05 using Tukey's test. Different superscript uppercase letters (within each row) show differences between the salinity stress levels within the same analysis group (*p* < .05). Different superscript lowercase letters (within each column) show differences between ecotypes within the same analysis day (*p* < .05).

**TABLE 3:** Content of chlorophyll b (µg/mL) related to different mint ecotypes and salinity stress levels at first and second harvest

Ecotypes	First harvest				Second harvest			
	Salt stress (dS/m)				Salt stress (dS/m)			
	0	2.5	5	7.5	0	2.5	5	7.5
E_1_	3.25 (±0.58) ^b‐d A^	3.09 (±0.58) ^b‐d A^	2.66 (±0.58) ^d‐f A^	2.44 (±0.58) ^c‐f A^	3.00 (±0.58) ^c‐e A^	3.07 (±0.58) ^b‐d A^	2.41 (±0.04) ^de A^	2.13 (±0.07) ^d‐h A^
E_2_	2.67 (±0.19) ^de A^	2.62 (±0.08) ^c‐g AB^	2.49 (±0.14) ^ef AB^	2.19 (±0.06) ^d‐g B^	2.45 (±0.17) ^de AB^	2.58 (±0.15) ^d‐h A^	2.45 (±0.25) ^de AB^	1.94 (±0.11) ^e‐i B^
E_3_	2.54 (±0.21) ^de A^	2.40 (±0.07) ^d‐g AB^	2.14 (±0.04) ^ef BC^	1.93 (±0.03) ^fg C^	2.49 (±0.24) ^de A^	2.29 (±0.24) ^e‐h AB^	1.92 (±0.10) ^ef AB^	1.80 (±0.11) ^f‐i B^
E_4_	2.64 (±0.24) ^de A^	2.50 (±0.04) ^c‐g A^	2.24 (±0.12) ^ef AB^	1.82 (±0.03) ^g B^	2.48 (±0.21) ^de A^	2.36 (±0.18) ^d‐h A^	2.23 (±0.19) ^de A^	1.52 (±0.03) ^i B^
E_5_	2.94 (±0.31) ^c‐e A^	2.85 (±0.26) ^c‐f A^	2.65 (±0.12) ^d‐f A^	2.47 (±0.06) ^c‐e A^	2.86 (±0.20) ^c‐e A^	2.82 (±0.07) ^c‐f AB^	2.38 (±)0.04 ^de BC^	2.24 (±0.18) ^c‐f C^
E_6_	2.42 (±0.25) ^de A^	2.29 (±0.17) ^e‐g AB^	2.03 (±0.21) ^fg AB^	1.96 (±0.08) ^e‐g B^	2.23 (±0.18) ^de A^	2.18 (±0.20) ^f‐h A^	1.97 (±0.09) ^d‐f A^	1.74 (±0.16) ^g‐i A^
E_7_	2.40 (±0.25) ^de A^	2.28 (±0.20) ^fg AB^	1.97 (±0.03) ^fg AB^	1.80 (±0.04) ^g B^	2.08 (±0.20) ^e A^	1.97 (±0.20) ^gh A^	1.92 (±0.12) ^ef A^	1.64 (±0.14) ^hi A^
E_8_	3.19 (±0.20) ^b‐e A^	3.02 (±0.23) ^b‐e AB^	2.59 (±0.12) ^d‐f BC^	2.46 (±0.04) ^c‐e C^	2.92 (±0.27) ^c‐e A^	2.84 (±0.30) ^c‐f A^	2.58 (±0.18) ^cd A^	2.34 (±0.15) ^b‐e A^
E_9_	3.36 (±0.27) ^b‐d A^	3.22 (±0.17) ^bc A^	2.79 (±0.19) ^c‐e AB^	2.55 (±0.04) ^cd B^	3.14 (±0.25) ^b‐d A^	3.03 (±0.23) ^b‐e AB^	2.54 (±0.16) ^c‐e BC^	2.41 (±0.13) ^b‐e C^
E_10_	3.20 (±0.24) ^b‐e A^	3.04 (±0.05) ^b‐d AB^	2.59 (±0.16) ^d‐f BC^	2.37 (±0.05) ^c‐f C^	2.89 (±0.25) ^c‐e A^	2.96 (±0.15) ^c‐e A^	2.45 (±0.24) ^de A^	2.36 (±0.22) ^b‐e A^
E_11_	2.48 (±0.20) ^de A^	2.36 (±0.22) ^d‐g A^	2.15 (±0.20) ^ef A^	1.98 (±0.06) ^e‐g A^	2.19 (±0.14) ^e A^	2.05 (±0.17) ^gh AB^	2.00 (±0.09) ^d‐f AB^	1.66 (±0.12) ^hi B^
E_12_	2.55 (±0.26) ^de A^	2.46 (±0.14) ^d‐g A^	2.19 (±0.23) ^ef A^	1.94 (±0.07) ^fg^	2.49 (±0.20) ^de A^	2.38 (±0.10) ^d‐h A^	2.18 (±0.23) ^de A^	1.62 (±0.15) ^i B^
E_13_	4.04 (±0.33) ^ab A^	3.96 (±0.11) ^a A^	3.55 (±0.10) ^ab AB^	3.11 (±0.14) ^ab B^	3.95 (±0.27) ^ab A^	3.90 (±0.11) ^a A^	3.53 (±0.18) ^ab A^	2.79 (±0.19) ^b B^
E_14_	3.69 (±0.36) ^a‐c A^	3.62 (±0.27) ^ab A^	3.18 (±0.22) ^b‐d AB^	2.73 (±0.13) ^bc B^	3.55 (±0.37) ^a‐c A^	3.34 (±0.14) ^a‐c A^	3.05 (±0.28) ^bc AB^	2.48 (±0.07) ^b‐d B^
E_15_	4.01 (±0.30) ^ab A^	3.89 (±0.38) ^a AB^	3.37 (±0.16) ^a‐c AB^	3.05 (±0.16) ^b B^	3.70 (±0.38) ^a‐c A^	3.71 (±0.19) ^ab A^	3.18 (±0.06) ^b AB^	2.73 (±0.27) ^bc B^
E_16_	2.20 (±0.20) ^e A^	1.94 (±0.07) ^g A^	1.46 (±0.14) ^g AB^	1.28 (±0.07) ^h B^	2.15 (±0.17) ^e A^	1.92 (±0.07) ^h A^	1.45 (±0.15) ^f B^	1.00 (±0.10) ^j C^
E_17_	2.89 (±0.23) ^c‐e A^	2.82 (±0.05) ^c‐f AB^	2.57 (±0.21) ^d‐f AB^	2.34 (±0.05) ^c‐f B^	2.62 (±0.26) ^de A^	2.70 (±0.25) ^c‐g A^	2.26 (±0.04) ^de A^	2.16 (±0.21) ^d‐j A^
E_18_	4.33 (±0.45) ^a A^	4.28 (±0.07) ^a A^	3.94 (±0.18) ^a A^	3.55 (±0.06) ^a A^	4.23 (±0.39) ^a A^	4.00 (±0.21) ^a A^	3.85 (±0.40) ^a A^	3.40 (±0.22) ^a A^
Species								
*longifolia*	2.82 (±0.11) ^bc A^	2.70 (±0.09) ^b A^	2.39 (±0.08) ^c B^	2.18 (±0.08) ^c B^	2.63 (±0.10) ^b A^	2.57 (±0.10) ^bc A^	2.26 (±0.06) ^c B^	1.97 (±0.06) ^c C^
*pulegium*	3.07 (±0.20) ^b A^	2.95 (±0.18) ^b A^	2.62 (±0.17) ^c AB^	2.35 (±0.13) ^c B^	2.88 (±0.20) ^b A^	2.82 (±0.19) ^b A^	2.54 (±0.18) ^c AB^	2.11 (±0.15) ^bc B^
*spicata*	3.85 (±0.23) ^a A^	3.76 (±0.22) ^a BC^	3.27 (±0.13) ^b BC^	2.89 (±0.11) ^b C^	3.63 (±0.25) ^a A^	3.53 (±0.13) ^a A^	3.11 (±0.14) ^b A^	2.61 (±0.14) ^b B^
*rotundifolia*	2.20 (±0.20) ^c A^	1.94 (±0.07) ^c A^	1.46 (±0.14) ^d B^	1.28 (±0.07) ^d B^	2.15 (±0.17) ^b A^	1.92 (±0.07) ^c A^	1.45 (±0.15) ^d B^	1.00 (±0.10) ^d C^
*mozafariani*	2.89 (±0.23) ^bc A^	2.82 (±0.05) ^b AB^	2.57 (±0.21) ^c AB^	2.34 (±0.05) ^c B^	2.62 (±0.26) ^b A^	2.70 (±0.25) ^b A^	2.26 (±0.04) ^c A^	2.16 (±0.21) ^bc A^
*piperita*	4.33 (±0.45) ^a A^	4.28 (±0.07) ^a A^	3.94 (±0.18) ^a A^	3.55 (±0.06) ^a A^	4.23 (±0.39) ^a A^	4.00 (±0.21) ^a A^	3.85 (±0.40) ^a A^	3.40 (±0.22) ^a A^
**Total mean**	**3.04 (±0.10) ^A^**	**2.92 (±0.09) ^A^**	**2.59 (±0.08) ^A^**	**2.33 (±0.07) ^A^**	**2.86 (±0.09) ^A^**	**2.78 (±0.09) ^A^**	**2.46 (±0.08) ^A^**	**2.11 (±0.07) ^B^**

Note

Results are represented as mean ± standard error. Means in a column and row followed by the different superscripts are significantly different at *p* ≤ .05 using Tukey's test. Different superscript uppercase letters (within each row) show differences between the salinity stress levels within the same analysis group (*p* < .05). Different superscript lowercase letters (within each column) show differences between ecotypes within the same analysis day (*p* < .05).

**TABLE 4:** Content of total chlorophyll (µg/mL) related to different mint ecotypes and salinity stress levels at first and second harvest

Ecotypes	First harvest				Second harvest			
	Salt stress (dS/m)				Salt stress (dS/m)			
	0	2.5	5	7.5	0	2.5	5	7.5
E_1_	11.61 (±1.07) ^cd A^	11.03 (±0.73) ^d A^	9.02 (±0.87) ^bc AB^	7.38 (±1.12) ^d‐f B^	11.41 (±1.08) ^d‐f A^	11.08 (±0.88) ^d A^	9.06 (±0.80) ^cd AB^	7.28 (±0.41) ^d‐f B^
E_2_	8.99 (±0.79) ^e‐g A^	8.50 (±0.19) ^fg AB^	7.60 (±0.42) ^c AB^	7.30 (±0.07) ^d‐f B^	8.87 (±0.26) ^gh A^	8.47 (±0.23) ^i AB^	7.72 (±0.74) ^de AB^	7.29 (±0.19) ^d‐f B^
E_3_	9.41 (±0.32) ^e‐g A^	8.66 (±0.25) ^fg A^	7.52 (±0.46) ^c B^	7.32 (±0.19) ^d‐f B^	9.48 (±0.57) ^f‐h A^	8.68 (±0.19) ^hi AB^	7.62 (±0.57) ^de B^	7.50 (±0.31) ^d‐f B^
E_4_	8.66 (±0.79) ^fg A^	8.16 (±0.12) ^g AB^	7.24 (±0.41) ^c AB^	6.82 (±0.11) ^f B^	8.80 (±0.41) ^gh A^	8.26 (±0.41) ^i AB^	7.26 (±0.53) ^de BC^	6.65 (±0.09) ^f C^
E_5_	10.83 (±0.32) ^de A^	10.35 (±0.43) ^de A^	8.82 (±0.48) ^bc B^	8.64 (±0.39) ^c B^	10.79 (±0.33) ^e‐g A^	10.33 (±0.14) ^d‐f A^	8.73 (±)0.48 ^c‐e B^	8.69 (±0.29) ^c B^
E_6_	8.45 (±0.39) ^g A^	8.07 (±0.37) ^g AB^	7.13 (±0.57) ^c B^	7.06 (±0.25) ^ef B^	8.39 (±0.74) ^h A^	8.23 (±0.20) ^i A^	7.08 (±0.46) ^e A^	7.05 (±0.48) ^ef A^
E_7_	8.87 (±0.36) ^e‐g A^	8.55 (±0.33) ^fg A^	7.36 (±0.48) ^c B^	7.19 (±0.17) ^d‐f B^	8.79 (±0.40) ^gh A^	8.39 (±0.22) ^i AB^	7.39 (±0.50) ^de BC^	7.23 (±0.15) ^d‐f C^
E_8_	11.73 (±0.38) ^cd A^	10.97 (±0.39) ^d A^	8.96 (±0.55) ^bc B^	8.83 (±0.31) ^c B^	11.68 (±0.38) ^c‐e A^	10.93 (±0.32) ^de A^	8.99 (±0.60) ^c‐e B^	8.95 (±0.46) ^c B^
E_9_	10.72 (±0.13) ^de A^	9.99 (±0.34) ^de A^	8.55 (±0.41) ^c B^	8.31 (±0.12) ^cd^	10.53 (±0.73) ^e‐g A^	9.81 (±0.35) ^e‐g AB^	8.33 (±0.66) ^de B^	8.40 (±0.44) ^cd B^
E_10_	10.45 (±0.37) ^d‐f A^	9.86 (±0.12) ^e A^	8.28 (±0.52) ^c B^	8.06 (±0.19) ^c‐e B^	10.47 (±0.74) ^e‐g A^	9.96 (±0.34) ^ef A^	8.38 (±0.26) ^de B^	8.25 (±0.42) ^c‐e B^
E_11_	8.95 (±0.78) ^e‐g A^	8.63 (±0.26) ^fg A^	7.51 (±0.36) ^c A^	7.33 (±0.36) ^d‐f A^	8.77 (±0.47) ^gh A^	8.37 (±0.18) ^i AB^	7.44 (±0.46) ^de BC^	7.16 (±0.11) ^ef C^
E_12_	9.13 (±0.60) ^e‐g A^	8.71 (±0.19) ^fg A^	7.48 (±0.33) ^c B^	7.22 (±0.21) ^d‐f B^	9.13 (±0.25) ^gh A^	8.74 (±0.18) ^g‐i AB^	7.62 (±0.64) ^de BC^	7.17 (±0.41) ^ef C^
E_13_	14.60 (±0.36) ^ab A^	14.41 (±0.50) ^ab A^	11.55 (±0.44) ^a B^	11.10 (±0.21) ^ab B^	14.69 (±0.29) ^ab A^	14.57 (±0.35) ^a A^	11.62 (±0.55) ^a B^	10.92 (±0.47) ^ab B^
E_14_	13.34 (±0.91) ^bc A^	12.98 (±0.29) ^c A^	10.50 (±0.80) ^ab B^	10.06 (±0.43) ^b B^	13.44 (±0.43) ^bc A^	13.01 (±0.37) ^bc A^	10.40 (±0.26) ^a‐c B^	10.02 (±0.11) ^b B^
E_15_	14.75 (±0.71) ^ab A^	13.99 (±0.32) ^b A^	11.00 (±0.61) ^a B^	10.68 (±0.38) ^ab B^	14.50 (±0.72) ^ab A^	13.96 (±0.45) ^ab A^	10.82 (±0.57) ^ab B^	10.64 (±0.47) ^ab B^
E_16_	12.76 (±0.71) ^c A^	12.08 (±0.33) ^c A^	9.02 (±0.82) ^bc B^	8.85 (±0.12) ^c B^	12.96 (±1.17) ^b‐d A^	12.20 (±0.40) ^c A^	9.13 (±0.56) ^b‐d B^	8.67 (±0.48) ^c B^
E_17_	10.25 (±0.33) ^d‐g A^	9.59 (±0.23) ^ef A^	8.12 (±0.59) ^c B^	7.88 (±0.11) ^c‐f B^	10.22 (±0.40) ^e‐h A^	9.61 (±0.22) ^f‐h A^	8.13 (±0.53) ^de BC^	7.84 (±0.27) ^c‐f B^
E_18_	15.56 (±0.48) ^a A^	15.06 (±0.33) ^a A^	11.94 (±0.91) ^a B^	11.55 (±0.09) ^a B^	15.73 (±0.46) ^a A^	14.89 (±0.35) ^a A^	12.13 (±0.76) ^a B^	11.48 (±0.47) ^a B^
Species								
*longifolia*	9.92 (±0.27) ^d A^	9.36 (±0.22) ^d A^	8.02 (±0.20) ^b B^	7.65 (±0.17) ^b B^	9.86 (±0.27) ^c A^	9.35 (±0.22) ^d A^	8.02 (±0.22) ^c B^	7.67 (±0.16) ^b B^
*pulegium*	10.78 (±0.64) ^cd A^	10.40 (±0.63) ^cd A^	8.70 (±0.47) ^b B^	8.43 (±0.42) ^b B^	10.76 (±0.64) ^c A^	10.41 (±0.65) ^cd A^	8.76 (±0.49) ^c B^	8.38 (±0.43) ^b B^
*spicata*	14.05 (±0.60) ^ab A^	13.49 (±0.28) ^ab A^	10.75 (±0.48) ^a B^	10.37 (±0.29) ^a B^	13.97 (±0.44) ^ab A^	13.49 (±0.32) ^ab A^	10.61 (±0.30) ^ab B^	10.33 (±0.25) ^a B^
*rotundifolia*	12.76 (±0.71) ^bc A^	12.08 (±0.33) ^bc A^	9.02 (±0.82) ^b B^	8.85 (±0.12) ^b B^	12.96 (±1.17) ^b A^	12.20 (±0.40) ^bc A^	9.13 (±0.56) ^bc B^	8.67 (±0.48) ^b B^
*mozafariani*	10.25 (±0.33) ^d A^	9.59 (±0.23) ^d A^	8.12 (±0.59) ^b B^	7.88 (±0.11) ^b B^	10.22 (±0.40) ^c A^	9.61 (±0.22) ^d A^	8.13 (±0.53) ^c B^	7.84 (±0.27) ^b B^
*piperita*	15.56 (±0.48) ^a A^	15.06 (±0.33) ^a A^	11.94 (±0.91) ^a B^	11.55 (±0.09) ^a B^	15.73 (±0.46) ^a A^	14.89 (±0.35) ^a A^	12.13 (±0.76) ^a B^	11.48 (±0.47) ^a B^
**Total mean**	**11.06 (±0.29) ^A^**	**10.53 (±0.27) ^A^**	**8.75 (±0.21) ^A^**	**8.42 (±0.19) ^A^**	**11.04 (±0.30) ^A^**	**10.53 (±0.27) ^A^**	**8.77 (±0.21) ^A^**	**8.40 (±0.19) ^A^**

Note

Results are represented as mean ± standard error. Means in a column and row followed by the different superscripts are significantly different at *p* ≤ .05 using Tukey's test. Different superscript uppercase letters (within each row) show differences between the salinity stress levels within the same analysis group (*p* < .05). Different superscript lowercase letters (within each column) show differences between ecotypes within the same analysis day (*p* < .05).

**TABLE 5:** Ratio of chlorophyll a/b related to different mint ecotypes and salinity stress levels at first and second harvest

Ecotypes	First harvest				Second harvest			
	Salt stress (dS/m)				Salt stress (dS/m)			
	0	2.5	5	7.5	0	2.5	5	7.5
E_1_	2.79 (±0.47) ^b A^	3.02 (±0.84) ^b A^	2.75 (±0.71) ^b A^	2.32 (±0.49) ^c A^	2.98 (±0.34) ^b A^	2.84 (±0.49) ^bc A^	2.77 (±0.22) ^b A^	2.42 (±0.27) ^de A^
E_2_	2.36 (±0.12) ^b A^	2.25 (±0.09) ^bc A^	2.08 (±0.24) ^b A^	2.34 (±0.12) ^bc A^	2.65 (±0.14) ^b AB^	2.30 (±0.10) ^c BC^	2.17 (±0.13) ^b C^	2.78 (±0.17) ^b‐e A^
E_3_	2.75 (±0.19) ^b A^	2.60 (±0.10) ^bc A^	2.52 (±0.17) ^b A^	2.79 (±0.16) ^bc A^	2.86 (±0.23) ^b A^	2.90 (±0.38) ^bc A^	3.02 (±0.40) ^b A^	3.20 (±0.30) ^b‐e A^
E_4_	2.31 (±0.23) ^b A^	2.27 (±0.08) ^bc A^	2.26 (±0.26) ^b A^	2.75 (±0.05) ^bc A^	2.66 (±0.42) ^b AB^	2.53 (±0.12) ^bc AB^	2.29 (±0.24) ^b B^	3.38 (±0.13) ^b‐d A^
E_5_	2.81 (±0.39) ^b A^	2.69 (±0.27) ^bc A^	2.35 (±0.22) ^b A^	2.51 (±0.22) ^bc A^	2.80 (±0.14) ^b A^	2.67 (±0.08) ^bc A^	2.67 (±0.20) ^b A^	2.93 (±0.22) ^b‐e A^
E_6_	2.64 (±0.46) ^b A^	2.56 (±0.14) ^bc A^	2.60 (±0.37) ^b A^	2.61 (±0.06) ^bc A^	2.78 (±0.19) ^b A^	2.83 (±0.25) ^bc A^	2.65 (±0.40) ^b A^	3.11 (±0.24) ^b‐e A^
E_7_	2.79 (±0.30) ^b A^	2.81 (±0.24) ^bc A^	2.74 (±0.22) ^b A^	3.01 (±0.15) ^b A^	3.40 (±0.61) ^b A^	3.39 (±0.40) ^b A^	2.91 (±0.40) ^b A^	3.52 (±0.42) ^bc A^
E_8_	2.70 (±0.14) ^b A^	2.66 (±0.16) ^bc A^	2.49 (±0.30) ^b A^	2.59 (±0.11) ^bc A^	3.08 (±0.32) ^b A^	2.99 (±0.47) ^bc A^	2.49 (±0.09) ^b A^	2.87 (±0.31) ^b‐e A^
E_9_	2.25 (±0.24) ^b A^	2.11 (±0.08) ^c A^	2.11 (±0.26) ^b A^	2.26 (±0.04) ^c A^	2.38 (±0.19) ^b A^	2.28 (±0.18) ^c A^	2.28 (±0.18) ^b A^	2.48 (±0.11) ^de A^
E_10_	2.30 (±0.20) ^b A^	2.24 (±0.06) ^bc A^	2.20 (±0.11) ^b A^	2.41 (±0.14) ^bc A^	2.69 (±0.37) ^b A^	2.37 (±0.07) ^c A^	2.53 (±0.39) ^b A^	2.56 (±0.28) ^c‐e A^
E_11_	2.63 (±0.19) ^b A^	2.73 (±0.24) ^bc A^	2.59 (±0.39) ^b A^	2.70 (±0.16) ^bc A^	3.07 (±0.39) ^b A^	3.16 (±0.28) ^bc A^	2.71 (±0.10) ^b A^	3.39 (±0.34) ^b‐d A^
E_12_	2.67 (±0.35) ^b A^	2.57 (±0.18) ^bc A^	2.55 (±0.44) ^b A^	2.74 (±0.16) ^bc A^	2.73 (±0.27) ^b AB^	2.69 (±0.13) ^bc AB^	2.54 (±0.25) ^b B^	3.53 (±0.41) ^b A^
E_13_	2.66 (±0.20) ^b A^	2.64 (±0.09) ^bc A^	2.26 (±0.20) ^b A^	2.59 (±0.13) ^bc A^	2.78 (±0.29) ^b A^	2.74 (±0.10) ^bc A^	2.31 (±0.19) ^b A^	2.95 (±0.21) ^b‐e A^
E_14_	2.66 (±0.22) ^b A^	2.63 (±0.19) ^bc A^	2.32 (±0.19) ^b A^	2.71 (±0.25) ^bc A^	2.93 (±0.47) ^b A^	2.90 (±0.10) ^bc A^	2.52 (±0.40) ^b A^	3.04 (±0.12) ^b‐e A^
E_15_	2.70 (±0.12) ^b A^	2.69 (±0.34) ^bc A^	2.28 (±0.22) ^b A^	2.53 (±0.21) ^bc A^	3.05 (±0.44) ^b A^	2.78 (±0.15) ^bc A^	2.40 (±0.19) ^b AB^	3.00 (±0.40) ^b‐e A^
E_16_	4.98 (±0.76) ^a A^	5.22 (±0.16) ^a A^	5.30 (±0.60) ^a A^	5.97 (±0.34) ^a A^	5.10 (±0.66) ^a B^	5.37 (±0.33) ^a B^	5.55 (±0.88) ^a B^	7.80 (±0.48) ^a A^
E_17_	2.59 (±0.18) ^b A^	2.41 (±0.10) ^bc A^	2.18 (±0.16) ^b A^	2.38 (±0.09) ^bc A^	2.98 (±0.28) ^b A^	2.66 (±0.37) ^bc A^	2.60 (±0.25) ^b A^	2.72 (±0.30) ^b‐e A^
E_18_	2.68 (±0.30) ^b A^	2.52 (±0.12) ^bc AB^	2.03 (±0.21) ^b B^	2.26 (±0.08) ^c AB^	2.82 (±0.38) ^b A^	2.74 (±0.12) ^bc A^	2.26 (±0.43) ^b A^	2.40 (±0.19) ^e A^
Species								
*longifolia*	2.60 (±0.10) ^b A^	2.55 (±0.10) ^b A^	2.43 (±0.11) ^b A^	2.58 (±0.07) ^b A^	2.84 (±0.10) ^b AB^	2.75 (±0.11) ^b AB^	2.58 (±0.09) ^b B^	2.97 (±0.10) ^b A^
*pulegium*	2.57 (±0.12) ^b A^	2.55 (±0.09) ^b A^	2.40 (±0.15) ^b A^	2.61 (±0.07) ^b A^	2.82 (±0.15) ^b AB^	2.74 (±0.10) ^b AB^	2.52 (±0.12) ^b B^	3.11 (±0.17) ^b A^
*spicata*	2.68 (±0.11) ^b A^	2.66 (±0.18) ^b A^	2.30 (±0.13) ^b A^	2.62 (±0.15) ^b A^	2.99 (±0.30) ^b A^	2.84 (±0.09) ^b A^	2.46 (±0.20) ^b A^	3.02 (±0.19) ^b A^
*rotundifolia*	4.98 (±0.76) ^a A^	5.22 (±0.16) ^a A^	5.30 (±0.60) ^a A^	5.97 (±0.34) ^a A^	5.10 (±0.66) ^a B^	5.37 (±0.33) ^a B^	5.55 (±0.88) ^a B^	7.80 (±0.48) ^a A^
*mozafariani*	2.59 (±0.18) ^b A^	2.41 (±0.10) ^b A^	2.18 (±0.16) ^b A^	2.38 (±0.09) ^b A^	2.98 (±0.28) ^b A^	2.66 (±0.37) ^b A^	2.60 (±0.25) ^b A^	2.72 (±0.30) ^b A^
*piperita*	2.68 (±0.30) ^b A^	2.52 (±0.12) ^b AB^	2.03 (±0.21) ^b B^	2.26 (±0.08) ^b AB^	2.82 (±0.38) ^b A^	2.74 (±0.12) ^b A^	2.26 (±0.43) ^b A^	2.40 (±0.19) ^b A^
**Total mean**	**2.74 (±0.09) ^B^**	**2.70 (±0.09) ^B^**	**2.53 (±0.11) ^A^**	**2.75 (±0.10) ^B^**	**2.99 (±0.10) ^A^**	**2.90 (±0.10) ^A^**	**2.70 (±0.11) ^A^**	**3.23 (±0.15) ^A^**

Note

Results are represented as mean ± standard error. Means in a column and row followed by the different superscripts are significantly different at *p* ≤ .05 using Tukey's test. Different superscript uppercase letters (within each row) show differences between the salinity stress levels within the same analysis group (*p* < .05). Different superscript lowercase letters (within each column) show differences between ecotypes within the same analysis day (*p* <.05).

Based on the results from the first harvest, the E18 had the highest amount of chlorophyll a in normal and salinity stress conditions (Table 2). The comparison between different salinity levels showed that in most ecotypes, salinity stress reduced chlorophyll a. Also, the results showed that there was no significant difference between the salinity levels in E2, E4, E6, E11, and E12 ecotypes. The results of chlorophyll a in the second harvest stage were different. In the second harvest, the normal level, E13, E15, E16, and E18 ecotypes had the highest content of chlorophyll a. At salinity levels of 2.5 dS/m, E13 and E18 ecotypes had the highest chlorophyll a. As the salinity stress increased to the level of 5 dS/m, only the E18 ecotype showed the highest chlorophyll a content. Interestingly, the ecotypes with the highest levels of chlorophyll a at normal condition also showed the highest levels of chlorophyll a at 7.5 dS/m stress level. In E3, E4, E6, E7, and E11 ecotypes, there was no significant difference between the different salinity levels. The highest content of chlorophyll a was found in the normal conditions in the second harvest, which did not significantly differ from other treatments. In contrast, the lowest chlorophyll a was related to the 7.5 dS/m salinity treatment in the first harvest. The results determination of chlorophyll b content in different treatment and ecotypes at the first and second harvest stage were presented in Table 3. At the first harvest, the highest and lowest levels of chlorophyll b were associated with E18 and E16 ecotypes, respectively.

Only at the 7.5 dS/m salinity level, a significant difference was observed between the two harvests stages, where the highest chlorophyll b content was found in the first harvest. Also, chlorophyll b content in E1, E5, E11, and E18 ecotypes was not affected by salinity stress. There results in the case of the second harvest were different. In the most of the ecotypes, chlorophyll b content did not decrease with increasing of the salinity stress. In the second harvest, E1, E6, E7, E8, E10, E17, and E18 ecotypes were not affected by the salinity stress at different levels.

The results for total chlorophyll content were presented in Table 4. In the first and second harvest stages, the control level, 2.5 and 5 dS/m, E18 and E6 ecotypes showed the highest and lowest total chlorophyll content, respectively. In addition, in both harvest stages at 7.5 dS/m salinity level, E18 and E4 had the highest and lowest total chlorophyll content, respectively. There was no significant difference between the two harvest stages at different salinity levels. Furthermore, in the first harvest stage, the total chlorophyll content was not affected by salinity stress only in E11. However, in the second harvest stage in E6, the total chlorophyll content was not affected by the salinity stress. Table 5 shows the comparative results of chlorophyll a/b ratio. In each harvest stages, the highest chlorophyll a/b ratio was found in E16. In contrast, the lowest chlorophyll a/b ratio in both harvest stages at 2.5 dS/m salinity level was observed in E9, while at 7.5 dS/m salinity level, the lowest chlorophyll a/b ratio was found in E18. There was a significant difference in chlorophyll a/b ratio between control, 2.5 and 7.5 dS/m levels. The results showed that the highest ratios of chlorophyll a/b at control, 2.5 and 7.5 dS/m levels were observed in the second harvest stage.

The highest content of carotenoids was observed in the absence of salinity stress, and with increasing salinity stress, the content of carotenoids decreased (Table 6). Comparison between different species showed that *piperita* and *rotundifolia* species had the highest and lowest carotenoids in all levels of salinity stress, respectively. Also, the amount of carotenoids in the second harvest had higher compared to the first harvest at all salinity stress levels. The results of the present experiment showed that in all levels of salinity stress, the highest content of total anthocyanin was observed in *piperita* species, while the lowest content of total anthocyanin was found in *rotundifolia* species (Table 7).

**TABLE 6:** Content of carotenoid (µg/mL) related to different mint ecotypes and salinity stress levels at first and second harvest

Ecotypes	First harvest				Second harvest			
	Salt stress (dS/m)				Salt stress (dS/m)			
	0	2.5	5	7.5	0	2.5	5	7.5
E_1_	1.54 (±0.58) ^a‐d A^	1.30 (±0.58) ^ab A^	1.23 (±0.58) ^a‐c A^	1.12 (±0.58) ^a‐c A^	1.79 (±0.06) ^b‐d A^	1.60 (±0.29) ^a‐c A^	1.35 (±0.58) ^a‐c A^	1.30 (±0.58) ^a‐d A^
E_2_	1.79 (±0.07) ^ab A^	1.69 (±0.09) ^a A^	1.66 (±0.15) ^ab A^	1.43 (±0.07) ^ab A^	1.92 (±0.13) ^a‐c A^	1.77 (±0.06) ^a A^	1.88 (±0.17) ^a A^	1.62 (±0.16) ^a‐d A^
E_3_	1.17 (±0.03) ^cd A^	1.03 (±0.02) ^bc AB^	0.97 (±0.09) ^cd B^	0.87 (±0.04) ^cd B^	1.36 (±0.13) ^c‐e A^	1.16 (±0.07) ^de AB^	1.17 (±0.03) ^cd AB^	1.09 (±0.03) ^cd B^
E_4_	1.47 (±0.03) ^a‐d A^	1.31 (±0.08) ^ab A^	1.23 (±0.11) ^a‐c A^	0.95 (±0.09) ^b‐d B^	1.73 (±0.16) ^b‐d A^	1.57 (±0.05) ^a‐c AB^	1.34 (±0.02) ^bc B^	1.01 (±0.05) ^cd C^
E_5_	1.26 (±0.02) ^cd A^	1.16 (±0.04) ^bc AB^	1.12 (±0.08) ^b‐d AB^	1.04 (±0.02) ^a‐d B^	1.27 (±0.08) ^de AB^	1.49 (±0.11) ^a‐d A^	1.42 (±0.10) ^a‐c AB^	1.16 (±0.02) ^b‐d B^
E_6_	1.65 (±0.06) ^a‐c A^	1.44 (±0.07) ^ab B^	1.36 (±0.03) ^a‐c B^	1.31 (±0.09) ^a‐c B^	1.77 (±0.13) ^b‐d A^	1.75 (±0.11) ^ab A^	1.38 (±0.03) ^a‐c B^	1.35 (±0.07) ^a‐c B^
E_7_	1.34 (±0.02) ^b‐d A^	1.13 (±0.05) ^bc B^	1.07 (±0.08) ^cd B^	0.97 (±0.03) ^b‐d B^	1.36 (±0.02) ^c‐e A^	1.45 (±0.09) ^a‐d A^	1.24 (±0.05) ^cd A^	1.22 (±0.08) ^b‐d A^
E_8_	1.34 (±0.03) ^b‐d A^	1.12 (±0.08) ^bc B^	1.05 (±0.08) ^cd B^	1.00 (±0.05) ^a‐d B^	1.52 (±0.06) ^c‐e A^	1.44 (±0.02) ^a‐d A^	1.11 (±0.08) ^cd B^	1.17 (±0.07) ^b‐d B^
E_9_	1.38 (±0.03) ^b‐d A^	1.17 (±0.02) ^bc B^	1.11 (±0.08) ^b‐d B^	1.01 (±0.06) ^a‐d B^	1.44 (±0.12) ^c‐e A^	1.27 (±0.13) ^c‐e A^	1.21 (±0.08) ^cd A^	1.29 (±0.10) ^a‐d A^
E_10_	1.42 (±0.03) ^a‐d A^	1.17 (±0.06) ^bc B^	1.11 (±0.03) ^b‐d B^	1.00 (±0.08) ^a‐d B^	1.59 (±0.05) ^c‐e A^	1.19 (±0.12) ^de B^	1.14 (±0.05) ^cd B^	1.04 (±0.02) ^cd B^
E_11_	1.45 (±0.03) ^a‐d A^	1.32 (±0.04) ^ab AB^	1.25 (±0.12) ^a‐c AB^	1.14 (±0.10) ^a‐c B^	1.75 (±0.11) ^b‐d A^	1.39 (±0.02) ^b‐d B^	1.53 (±0.06) ^a‐c B^	1.45 (±0.03) ^a‐c B^
E_12_	1.46 (±0.03) ^a‐d A^	1.29 (±0.09) ^ab AB^	1.24 (±0.11) ^a‐c AB^	1.08 (±0.08) ^a‐d B^	2.29 (±0.69) ^ab A^	1.46 (±0.03) ^a‐d AB^	1.48 (±0.14) ^a‐c AB^	1.09 (±0.07) ^cd B^
E_13_	1.54 (±0.06) ^a‐d A^	1.37 (±0.08) ^ab AB^	1.34 (±0.08) ^a‐c AB^	1.15 (±0.03) ^a‐c B^	1.71 (±0.04) ^b‐d A^	1.51 (±0.03) ^a‐d B^	1.48 (±0.07) ^a‐c B^	1.23 (±0.06) ^b‐d C^
E_14_	1.62 (±0.06) ^a‐d A^	1.39 (±0.08) ^ab B^	1.37 (±0.08) ^a‐c B^	1.14 (±0.07) ^a‐c C^	1.73 (±0.10) ^b‐d A^	1.61 (±0.08) ^a‐c AB^	1.51 (±0.08) ^a‐c AB^	1.37 (±0.03) ^a‐c B^
E_15_	1.37 (±0.02) ^b‐d A^	1.16 (±0.05) ^bc B^	1.12 (±0.03) ^b‐d B^	1.00 (±0.03) ^a‐d C^	1.69 (±0.12) ^b‐d A^	1.43 (±0.14) ^a‐d AB^	1.22 (±0.10) ^cd B^	1.32 (±0.03) ^a‐c B^
E_16_	1.15 (±0.05) ^d A^	0.76 (±0.04) ^c B^	0.66 (±0.02) ^d B^	0.57 (±0.01) ^d C^	1.03 (±0.07) ^e A^	1.02 (±0.04) ^e A^	0.77 (±0.05) ^d B^	0.80 (±0.06) ^d B^
E_17_	1.58 (±0.04) ^a‐d A^	1.43 (±0.08) ^ab AB^	1.39 (±0.05) ^a‐c AB^	1.26 (±0.12) ^a‐c B^	1.89 (±0.17) ^a‐d A^	1.47 (±0.08) ^a‐d B^	1.41 (±0.09) ^a‐c B^	1.28 (±0.13) ^a‐d B^
E_18_	1.89 (±0.05) ^a A^	1.73 (±0.03) ^a A^	1.71 (±0.17) ^a A^	1.52 (±0.13) ^a A^	2.39 (±0.04) ^a A^	1.74 (±0.05) ^ab B^	1.83 (±0.05) ^ab B^	1.78 (±0.05) ^a B^
Species								
*longifolia*	1.44 (±0.07) ^bc A^	1.26 (±0.07) ^b AB^	1.20 (±0.07) ^b B^	1.08 (±0.07) ^b B^	1.57 (±0.05) ^b A^	1.50 (±0.05) ^ab AB^	1.35 (±0.07) ^b BC^	1.24 (±0.07) ^b C^
*pulegium*	1.47 (±0.02) ^bc A^	1.29 (±0.04) ^b B^	1.23 (±0.05) ^b B^	1.09 (±0.04) ^b C^	1.84 (±0.17) ^b A^	1.39 (±0.04) ^b B^	1.41 (±0.06) ^b B^	1.20 (±0.05) ^b B^
*spicata*	1.49 (±0.06) ^bc A^	1.27 (±0.06) ^b B^	1.24 (±0.06) ^b B^	1.07 (±0.04) ^b C^	1.71 (±0.07) ^b A^	1.52 (±0.08) ^ab AB^	1.36 (±0.08) ^b B^	1.35 (±0.02) ^b B^
*rotundifolia*	1.15 (±0.05) ^c A^	0.76 (±0.04) ^c B^	0.66 (±0.02) ^c C^	0.57 (±0.01) ^c C^	1.03 (±0.07) ^c A^	1.02 (±0.04) ^c A^	0.77 (±0.05) ^c B^	0.80 (±0.06) ^c B^
*mozafariani*	1.58 (±0.04) ^ab A^	1.43 (±0.08) ^ab AB^	1.39 (±0.05) ^ab AB^	1.26 (±0.12) ^ab B^	1.89 (±0.17) ^b A^	1.47 (±0.08) ^ab B^	1.41 (±0.09) ^b B^	1.28 (±0.13) ^b B^
*piperita*	1.89 (±0.05) ^a A^	1.73 (±0.03) ^a AB^	1.71 (±0.17) ^a AB^	1.52 (±0.13) ^a B^	2.39 (±0.04) ^a A^	1.74 (±0.05) ^a B^	1.83 (±0.05) ^a B^	1.78 (±0.05) ^a B^
**Total mean**	**1.47 (±0.04) ^B^**	**1.27 (±0.04) ^B^**	**1.22 (±0.04) ^B^**	**1.09 (±0.04) ^B^**	**1.68 (±0.05) ^A^**	**1.46 (±0.03) ^A^**	**1.36 (±0.04) ^A^**	**1.25 (±0.04) ^A^**

Note

Results are represented as mean ± standard error. Means in a column and row followed by the different superscripts are significantly different at *p* ≤ .05 using Tukey's test. Different superscript uppercase letters (within each row) show differences between the salinity stress levels within the same analysis group (*p* < .05). Different superscript lowercase letters (within each column) show differences between ecotypes within the same analysis day (*p* < .05).

**TABLE 7:** Content of total anthocyanin (mg/g fresh weight) related to different mint ecotypes and salinity stress levels at first and second harvest

Ecotypes	First harvest				Second harvest			
	Salt stress (dS/m)				Salt stress (dS/m)			
	0	2.5	5	7.5	0	2.5	5	7.5
E_1_	3.19 (±0.30) ^ab C^	3.79 (±0.11) ^bc BC^	4.66 (±0.36) ^b‐d B^	6.00 (±0.24) ^bc A^	3.21 (±0.26) ^bc D^	3.85 (±0.08) ^bc C^	4.94 (±0.03) ^bc B^	6.16 (±0.27) ^bc A^
E_2_	3.42 (±0.18) ^a C^	4.18 (±0.02) ^ab C^	5.14 (±0.42) ^bc B^	6.15 (±0.27) ^bc A^	3.47 (±0.14) ^b D^	4.38 (±0.08) ^a‐c C^	5.42 (±0.18) ^ab B^	6.87 (±0.17) ^b A^
E_3_	3.29 (±0.13) ^ab C^	3.71 (±0.08) ^bc C^	4.81 (±0.29) ^b‐d B^	6.07 (±0.25) ^bc A^	3.51 (±0.34) ^b C^	3.90 (±0.20) ^bc C^	5.25 (±0.13) ^a‐c B^	6.29 (±0.15) ^bc A^
E_4_	3.51 (±0.14) ^a B^	3.90 (±0.29) ^b BC^	4.98 (±0.52) ^b‐d A^	5.73 (±0.21) ^bc A^	3.41 (±0.28) ^bc C^	4.19 (±0.24) ^bc C^	5.16 (±0.36) ^a‐c B^	6.45 (±0.22) ^bc A^
E_5_	3.29 (±0.14) ^ab C^	3.92 (±0.39) ^b BC^	4.93 (±0.26) ^b‐d B^	6.16 (±0.39) ^bc A^	3.39 (±0.12) ^bc D^	4.14 (±0.22) ^bc C^	5.03 (±0.32) ^a‐c B^	6.25 (±0.22) ^bc A^
E_6_	3.13 (±0.18) ^ab C^	4.11 (±0.24) ^ab B^	4.83 (±0.20) ^b‐d B^	6.35 (±0.44) ^b A^	3.38 (±0.31) ^bc C^	4.37 (±0.10) ^a‐c B^	5.12 (±0.24) ^a‐c B^	6.62 (±0.27) ^bc A^
E_7_	3.34 (±0.15) ^ab C^	3.92 (±0.20) ^b C^	5.05 (±0.23) ^b‐d B^	6.20 (±0.29) ^bc A^	3.47 (±0.12) ^b C^	4.06 (±0.23) ^bc C^	5.24 (±0.30) ^a‐c B^	6.41 (±0.26) ^bc A^
E_8_	3.23 (±0.32) ^ab C^	4.18 (±0.10) ^ab B^	4.50 (±0.07) ^cd B^	6.44 (±0.11) ^b A^	3.26 (±0.13) ^bc C^	4.51 (±0.31) ^ab B^	5.01 (±0.26) ^bc B^	6.60 (±0.19) ^bc A^
E_9_	3.15 (±0.29) ^ab C^	4.08 (±0.14) ^ab B^	4.81 (±0.19) ^b‐d B^	6.35 (±0.40) ^b A^	3.41 (±0.16) ^bc C^	4.34 (±0.33) ^a‐c B^	4.86 (±0.14) ^bc B^	6.52 (±0.34) ^bc A^
E_10_	3.18 (±0.15) ^ab C^	4.01 (±0.32) ^ab BC^	4.90 (±0.40) ^b‐d B^	6.28 (±0.36) ^b A^	3.24 (±0.11) ^bc D^	4.29 (±0.10) ^a‐c C^	4.91 (±0.14) ^bc B^	6.45 (±0.26) ^bc A^
E_11_	3.25 (±0.13) ^ab C^	4.20 (±0.15) ^ab B^	4.94 (±0.26) ^b‐d B^	6.47 (±0.37) ^b A^	3.26 (±0.08) ^bc C^	4.45 (±0.10) ^a‐c B^	4.54 (±0.19) ^cd B^	6.63 (±0.34) ^bc A^
E_12_	3.32 (±0.35) ^ab C^	4.01 (±0.07) ^ab BC^	5.03 (±0.38) ^b‐d B^	6.28 (±0.54) ^b A^	3.61 (±0.13) ^b B^	4.24 (±0.07) ^a‐c B^	5.36 (±0.56) ^a‐c A^	6.30 (±0.33) ^bc A^
E_13_	3.25 (±0.17) ^ab C^	4.14 (±0.09) ^ab BC^	4.94 (±0.40) ^b‐d B^	6.40 (±0.55) ^b A^	3.34 (±0.25) ^bc C^	4.44 (±0.41) ^a‐c B^	5.12 (±0.12) ^a‐c B^	6.44 (±0.15) ^bc A^
E_14_	3.32 (±0.23) ^ab D^	3.95 (±0.07) ^ab C^	5.56 (±0.09) ^ab B^	6.23 (±0.11) ^bc A^	3.44 (±0.12) ^bc C^	4.08 (±0.26) ^bc C^	5.03 (±0.35) ^a‐c B^	6.41 (±0.18) ^bc A^
E_15_	3.20 (±0.26) ^ab D^	3.95 (±0.18) ^ab C^	4.90 (±0.23) ^b‐d B^	6.35 (±0.22) ^b A^	3.28 (±0.15) ^bc D^	4.09 (±0.09) ^bc C^	5.11 (±0.15) ^a‐c B^	6.49 (±0.22) ^bc A^
E_16_	2.69 (±0.05) ^b D^	3.25 (±0.05) ^c C^	4.11 (±0.11) ^d B^	5.14 (±0.14) ^c A^	2.82 (±0.14) ^c C^	3.73 (±0.23) ^c B^	3.96 (±0.14) ^d B^	5.87 (±0.06) ^c A^
E_17_	3.26 (±0.15) ^ab D^	4.01 (±0.17) ^ab C^	4.94 (±0.11) ^b‐d B^	6.29 (±0.36) ^b A^	3.52 (±0.06) ^b C^	4.35 (±0.20) ^a‐c B^	4.89 (±0.19) ^bc B^	6.49 (±0.26) ^bc A^
E_18_	3.75 (±0.11) ^a D^	4.55 (±0.07) ^a C^	6.07 (±0.09) ^a B^	8.04 (±0.26) ^a A^	4.32 (±0.17) ^a D^	4.91 (±0.09) ^a C^	5.84 (±0.09) ^a B^	7.70 (±0.16) ^a A^
Species								
*longifolia*	3.28 (±0.07) ^b D^	3.98 (±0.07) ^b C^	4.86 (±0.10) ^b B^	6.16 (±0.10) ^b A^	3.39 (±0.07) ^b D^	4.19 (±0.07) ^bc C^	5.11 (±0.08) ^b B^	6.46 (±0.08) ^b A^
*pulegium*	3.25 (±0.10) ^b D^	4.09 (±0.09) ^b C^	4.95 (±0.16) ^b B^	6.36 (±0.21) ^b A^	3.36 (±0.08) ^b D^	4.36 (±0.10) ^b C^	4.98 (±0.16) ^b B^	6.46 (±0.13) ^b A^
*spicata*	3.26 (±0.16) ^b D^	3.95 (±0.09) ^b C^	5.23 (±0.17) ^b B^	6.29 (±0.11) ^b A^	3.36 (±0.09) ^b D^	4.08 (±0.13) ^bc C^	5.07 (±0.18) ^b B^	6.45 (±0.14) ^b A^
*rotundifolia*	2.69 (±0.05) ^c D^	3.25 (±0.05) ^c C^	4.11 (±0.11) ^c B^	5.14 (±0.14) ^c A^	2.82 (±0.14) ^c C^	3.73 (±0.23) ^c B^	3.96 (±0.14) ^c B^	5.87 (±0.06) ^c A^
*mozafariani*	3.26 (±0.15) ^b D^	4.01 (±0.17) ^b C^	4.94 (±0.11) ^b B^	6.29 (±0.36) ^b A^	3.52 (±0.06) ^b C^	4.35 (±0.20) ^b B^	4.89 (±0.19) ^b B^	6.49 (±0.26) ^b A^
*piperita*	3.75 (±0.11) ^a D^	4.55 (±0.07) ^a C^	6.07 (±0.09) ^a B^	8.04 (±0.26) ^a A^	4.32 (±0.17) ^a D^	4.91 (±0.09) ^a C^	5.84 (±0.09) ^a B^	7.70 (±0.16) ^a A^
**Total mean**	**3.27 (±0.05) ^B^**	**3.99 (±0.05) ^B^**	**4.95 (±0.07) ^A^**	**6.27 (±0.09) ^B^**	**3.41 (±0.05) ^A^**	**4.24 (±0.05) ^A^**	**5.04 (±0.07) ^A^**	**6.50 (±0.06) ^A^**

Note

Results are represented as mean ± standard error. Means in a column and row followed by the different superscripts are significantly different at *p* ≤.05 using Tukey's test. Different superscript uppercase letters (within each row) show differences between the salinity stress levels within the same analysis group (*p* <.05). Different superscript lowercase letters (within each column) show differences between ecotypes within the same analysis day (*p* <.05).

The highest and lowest levels of carotenoid content in both harvest stages were found in E18 and E16 ecotypes, respectively. Also, the highest content of carotenoid in all salinity levels was observed in the second harvest stage. Furthermore, the content of carotenoids in E1, E2, and E18 ecotypes was not affected by salinity stress in the first harvest stage. However, increasing the salinity stress in E1, E2, E7, and E9 ecotypes at the second harvest did not affect the carotenoid content.

According to Table 7, the highest and lowest contents of total anthocyanins at all stress levels (in both stages) were found in E18 and E16 ecotypes, respectively.

The results showed that the control, 2.5 and 7.5 dS/m in the first harvest had the lowest content of total anthocyanin. In the second harvest, total anthocyanins contents were higher. In general, the results showed that the content of total anthocyanins was affected by the salinity stress. The results revealed that in all ecotypes and at both harvest stages, the total anthocyanins content increased with increasing the salinity stress.

Photosynthetic pigments are important sources of photosynthetic products in plants. On the other hand, the efficiency and maintenance of photosynthetic performance in the face of plants with salinity stress conditions is one of the important topics in plant physiology and can play an important role in detecting salt sensitive and tolerant ecotypes (Dias et al., 2019; Gong et al., 2018; Melo et al., 2017; Siddiqi et al., 2009).

The results of this study showed that salinity stress had a negative effect on the content of photosynthetic pigments including chlorophyll a, b, total, and carotenoids. Salinity stress reduces the synthesis of chlorophyll a, b, and carotenoids, as well as the protein bonds, which may lead to a reduction in the potential of light absorption in the photosynthetic pigments (Huang et al., 2015; Tanveer et al., 2018; Wu et al., 2018). Under the salinity stress, ethylene and consequently chlorophyllase synthesis increases which cause the breakdown of chlorophyll (Trebitsh et al., 1993). It is noteworthy that in the present experiment, the content of photosynthetic pigments in some ecotypes was not affected by the salinity stress. Chlorophyllase appears to be less active in these ecotypes (Kaur et al., 2016; Rady, 2012). It has been also shown that chlorophyll enzyme activity is lower in tolerant ecotypes (Hanin et al., 2016). Other reasons for the decrease in chlorophyll in the plants include activation of chlorophyll catabolic pathway and lack of chlorophyll synthesis (Santos, 2004). In contrast, the total anthocyanins content increased with increasing the salinity stress. Under salinity stress condition, plants appear to increase their total anthocyanins content to protect themselves against stress damage (Kim et al., 2017). An increase in the synthesis of anthocyanins has been previously demonstrated in several plant species under the salinity stress condition including mint (Khalvandi et al., 2019), radish (Sakamoto & Suzuki, 2019) and canola (Kim et al., 2017). The results of our study showed that tolerant ecotypes have more total anthocyanin in contrast to sensitive ecotypes. The results of the present experiment revealed that in the control treatment, total anthocyanins content has a direct and significant correlation with antioxidant activity and essential oil amount. However, under the salt stress condition, the contents of carotenoids, chlorophyll b, and total chlorophyll showed a significant correlation with total anthocyanins content. At normal levels, high amount of total anthocyanin is not required to protect the light from the photosynthetic system due to the lack of stress. However, due to the reduction of chlorophyll a function, the role of chlorophyll b and carotenoids as auxiliary pigments in transferring the energy received to chlorophyll a and also compensating chlorophyll a deficiency were of great importance. A number of studies have pointed the role of chlorophyll b and carotenoids as auxiliary photosynthetic pigments in salinity stress and have shown that auxiliary pigments can play an effective role in the performance of photosynthetic pigments in salinity stress (Hashimoto et al., 2016; Kalteh et al., 2018).

In addition, several studies have shown that carotenoids often play a major role in protection against the light (Langi et al., 2018; Stahl & Sies, 2003; Young, 1991). Carotenoids are nonenzymatic antioxidants which provide optical protection by rapidly degrading the chlorophyll‐induced state. The induced state of the carotenoid lacks the energy needed to form oxygen radicals and therefore returns to its original state while losing energy in the form of heat. Carotenoids act as competitive inhibitors in the formation of oxygen radicals, and this protection process is vital for the plant, especially when light intensity increases drastically (Hashimoto et al., 2016; Havaux, 2014; Pandhair & Sekhon, 2006; Young, 1991).

Several studies have revealed that the production of total phenolic compounds increases with the salinity stress (Khalvandi et al., 2019; Taïbi et al., 2016; Yuan et al., 2010). Since the synthesis of total anthocyanins is derived from total phenolic compounds (Tanaka et al., 2008), the production of higher levels of total anthocyanins under the salinity stress condition is consistent with the results of the present experiment. Anthocyanin is a nonphotosynthetic pigment that has antioxidant activity by donating hydrogen, chelating metals, and binding proteins. Anthocyanins are able to keep the water potential constant, and therefore, plant tissues with higher anthocyanins are usually more resistant to stress (Edreva, 2005; khalvandi et al., 2019; Kovinich et al., 2015; Martín et al., 2017).

### 3.2 **Total** phenolic **and flavonoid content and antioxidant activity**

Based on the obtained results, it was observed that the total phenol and flavonoid content and antioxidant activity in different species was affected by the salinity stress, so that with increasing salinity stress, the amount of total phenol and flavonoid content and antioxidant activity was increased in all species (Tables 8, 9, and 10). The results showed that the highest and lowest amounts of total phenol and flavonoid content and antioxidant activity were found in *piperita* and *rotundifolia* species, respectively.

**TABLE 8:** Total phenolic content (mg/g) related to different mint ecotypes and salinity stress levels at first and second harvest

Ecotypes	First harvest				Second harvest			
	Salt stress (dS/m)				Salt stress (dS/m)			
	0	2.5	5	7.5	0	2.5	5	7.5
E_1_	9.53 (±0.58) ^bc D^	14.37 (±0.58) ^b‐d C^	19.53 (±0.58) ^b B^	22.55 (±0.58) ^b A^	9.58 (±0.58) ^bc D^	14.68 (±0.58) ^b C^	19.72 (±0.58) ^a B^	22.88 (±0.58) ^b A^
E_2_	10.01 (±0.35) ^b D^	15.66 (±0.45) ^a‐c C^	19.23 (±0.97) ^b B^	22.18 (±0.38) ^b A^	9.93 (±0.30) ^b C^	16.32 (±0.56) ^ab B^	20.11 (±0.70) ^b A^	22.36 (±1.22) ^bc A^
E_3_	9.28 (±0.16) ^bc D^	13.65 (±0.55) ^cd C^	19.28 (±0.78) ^b B^	21.89 (±0.88) ^b A^	9.56 (±0.33) ^bc D^	14.61 (±0.77) ^b C^	19.42 (±0.45) ^b B^	22.04 (±1.06) ^bc A^
E_4_	9.45 (±0.22) ^bc D^	14.26 (±0.74) ^b‐d C^	19.45 (±0.41) ^b B^	21.87 (±0.76) ^b A^	9.67 (±0.28) ^bc C^	14.40 (±1.16) ^b B^	19.64 (±0.34) ^b A^	21.50 (±0.29) ^bc A^
E_5_	9.42 (±0.27) ^bc D^	14.79 (±0.85) ^b‐d C^	19.43 (±0.58) ^b B^	22.34 (±0.65) ^b A^	9.72 (±0.45) ^bc C^	15.58 (±1.24) ^b B^	19.63 (±0.91) ^b A^	22.27 (±1.22) ^bc A^
E_6_	9.69 (±0.39) ^bc C^	14.37 (±0.91) ^b‐d B^	19.94 (±0.76) ^b A^	21.77 (±0.75) ^b A^	9.50 (±0.18) ^bc C^	14.55 (±1.43) ^b B^	19.92 (±0.58) ^b A^	21.97 (±1.27) ^bc A^
E_7_	9.87 (±0.23) ^b D^	14.58 (±0.67) ^b‐d C^	19.59 (±0.27) ^b B^	22.25 (±1.03) ^b A^	9.89 (±0.29) ^bc C^	14.72 (±1.11) ^b B^	20.11 (±0.70) ^b A^	22.00 (±0.82) ^bc A^
E_8_	9.48 (±0.16) ^bc D^	13.66 (±0.79) ^cd C^	19.48 (±0.56) ^b B^	22.62 (±0.91) ^ab A^	9.26 (±0.08) ^bc C^	13.76 (±1.19) ^bc B^	19.69 (±0.57) ^b A^	22.70 (±2.10) ^b A^
E_9_	9.38 (±0.22) ^bc D^	16.33 (±0.85) ^ab C^	19.38 (±1.79) ^b B^	22.66 (±0.52) ^ab A^	9.59 (±0.44) ^bc D^	16.48 (±1.43) ^ab C^	19.47 (±0.34) ^b B^	23.69 (±0.72) ^b A^
E_10_	9.53 (±0.17) ^bc D^	15.99 (±0.74) ^a‐c C^	19.33 (±0.46) ^b B^	22.63 (±0.91) ^ab A^	9.58 (±0.28) ^bc D^	16.14 (±1.58) ^ab C^	19.59 (±0.45) ^b B^	22.76 (±0.52) ^b A^
E_11_	9.47 (±0.33) ^bc D^	15.75 (±0.27) ^a‐c C^	19.27 (±0.49) ^b B^	21.74 (±0.88) ^b A^	9.80 (±0.23) ^bc C^	16.06 (±1.30) ^ab B^	19.62 (±0.79) ^b AB^	21.94 (±1.77) ^bc A^
E_12_	9.55 (±0.39) ^bc C^	15.33 (±0.27) ^a‐c B^	19.55 (±1.47) ^b A^	21.95 (±0.63) ^b A^	9.60 (±0.39) ^bc C^	15.54 (±1.35) ^b B^	19.74 (±0.57) ^b A^	22.22 (±1.67) ^bc A^
E_13_	9.99 (±0.23) ^b D^	15.48 (±0.89) ^a‐c C^	19.99 (±0.81) ^b B^	23.81 (±0.96) ^ab A^	10.08 (±0.29) ^b D^	16.44 (±0.92) ^ab C^	19.55 (±0.18) ^b B^	23.92 (±1.66) ^b A^
E_14_	9.65 (±0.17) ^bc D^	14.98 (±0.52) ^a‐d C^	19.65 (±1.59) ^b B^	23.11 (±0.40) ^ab A^	9.35 (±0.16) ^bc D^	15.82 (±1.15) ^b C^	18.94 (±0.23) ^bc B^	23.01 (±0.59) ^b A^
E_15_	9.42 (±0.18) ^bc D^	14.37 (±1.08) ^b‐d C^	19.42 (±1.57) ^b B^	23.39 (±0.68) ^ab A^	9.58 (±0.22) ^bc D^	15.10 (±0.58) ^b C^	19.63 (±0.41) ^b B^	22.03 (±0.78) ^bc A^
E_16_	8.77 (±0.09) ^c C^	12.66 (±0.51) ^d B^	17.11 (±0.47) ^b A^	19.49 (±0.66) ^b A^	8.76 (±0.32) ^c D^	11.10 (±0.31) ^c C^	17.64 (±0.22) ^c B^	18.82 (±0.59) ^c A^
E_17_	10.05 (±0.17) ^b D^	15.37 (±0.53) ^a‐c C^	20.05 (±0.38) ^b B^	22.67 (±0.92) ^ab A^	10.15 (±0.35) ^b C^	14.28 (±0.75) ^b B^	20.32 (±0.47) ^b A^	21.58 (±0.67) ^bc A^
E_18_	11.56 (±0.16) ^a C^	17.36 (±1.10) ^a B^	24.62 (±0.87) ^a A^	25.07 (±0.33) ^a A^	11.37 (±0.42) ^a D^	19.26 (±0.41) ^a C^	23.27 (±0.41) ^b B^	28.28 (±0.38) ^a A^
Species								
*longifolia*	9.57 (±0.10) ^b D^	14.63 (±0.25) ^b C^	19.48 (±0.25) ^b B^	22.24 (±0.22) ^b A^	9.64 (±0.11) ^b D^	15.01 (±0.35) ^b C^	19.75 (±0.18) ^b B^	22.38 (±0.35) ^b A^
*pulegium*	9.64 (±0.14) ^b D^	15.63 (±0.28) ^b C^	19.53 (±0.41) ^b B^	22.53 (±0.44) ^b A^	9.77 (±0.14) ^b D^	16.04 (±0.59) ^b C^	19.62 (±0.24) ^b B^	22.71 (±0.70) ^b A^
*spicata*	9.54 (±0.12) ^b D^	14.68 (±0.57) ^b C^	19.54 (±1.03) ^b B^	23.25 (±0.37) ^b A^	9.47 (±0.13) ^bc D^	15.46 (±0.61) ^b C^	19.29 (±0.25) ^b B^	22.52 (±0.49) ^b A^
*rotundifolia*	8.77 (±0.09) ^c D^	12.66 (±0.51) ^c C^	17.11 (±0.47) ^c B^	19.49 (±0.66) ^c A^	8.76 (±0.32) ^c C^	11.10 (±0.31) ^c B^	17.64 (±0.22) ^c A^	18.82 (±0.59) ^c A^
*mozafariani*	10.05 (±0.17) ^b D^	15.37 (±0.53) ^b C^	20.05 (±0.38) ^b B^	22.67 (±0.92) ^b A^	10.15 (±0.35) ^b C^	14.28 (±0.75) ^b B^	20.32 (±0.47) ^b A^	21.58 (±0.67) ^b A^
*piperita*	11.56 (±0.16) ^a C^	17.36 (±1.10) ^a B^	24.62 (±0.87) ^a A^	25.07 (±0.33) ^a A^	11.37 (±0.42) ^a D^	19.26 (±0.41) ^a C^	23.27 (±0.41) ^a B^	28.28 (±0.38) ^a A^
**Total mean**	**9.67 (±0.09) ^A^**	**14.94 (±0.20) ^A^**	**19.68 (±0.25) ^A^**	**22.44 (±0.20) ^A^**	**9.72 (±0.09) ^A^**	**15.27 (±0.29) ^A^**	**19.78 (±0.16) ^A^**	**22.55 (±0.31) ^A^**

Note

Results are represented as mean ± standard error. Means in a column and row followed by the different superscripts are significantly different at *p* ≤ .05 using Tukey's test. Different superscript uppercase letters (within each row) show differences between the salinity stress levels within the same analysis group (*p* < .05). Different superscript lowercase letters (within each column) show differences between ecotypes within the same analysis day (*p* < .05).

**TABLE 9:** Total flavonoid content (mg/g) related to different mint ecotypes and salinity stress levels at first and second harvest

Ecotypes	First harvest				Second harvest			
	Salt stress (dS/m)				Salt stress (dS/m)			
	0	2.5	5	7.5	0	2.5	5	7.5
E_1_	4.45 (±0.24) ^b C^	5.24 (±0.20) ^bc BC^	5.84 (±0.12) ^b B^	7.65 (±0.58) ^a‐c A^	4.19 (±0.58) ^bc B^	4.94 (±0.08) ^bc B^	6.34 (±0.22) ^b A^	7.44 (±0.58) ^a‐c A^
E_2_	4.47 (±0.13) ^b C^	5.45 (±0.06) ^b BC^	5.93 (±0.24) ^b B^	7.05 (±0.41) ^bc A^	4.19 (±0.36) ^bc C^	5.31 (±0.46) ^bc BC^	6.19 (±0.36) ^b AB^	6.91 (±0.40) ^bc A^
E_3_	4.87 (±0.39) ^b C^	5.54 (±0.15) ^b BC^	6.34 (±0.12) ^ab AB^	7.32 (±0.63) ^a‐c A^	4.85 (±0.28) ^b C^	5.28 (±0.40) ^bc BC^	6.08 (±0.11) ^b AB^	7.03 (±0.37) ^bc A^
E_4_	4.33 (±0.35) ^b C^	5.33 (±0.15) ^bc C^	6.45 (±0.48) ^ab B^	7.58 (±0.20) ^a‐c A^	4.15 (±0.29) ^bc C^	5.19 (±0.12) ^bc B^	6.18 (±0.18) ^b A^	6.63 (±0.31) ^c A^
E_5_	4.70 (±0.30) ^b C^	5.63 (±0.23) ^b BC^	6.48 (±0.52) ^ab AB^	7.33 (±0.55) ^a‐c A^	4.42 (±0.15) ^bc C^	5.38 (±0.53) ^bc BC^	6.22 (±0.57) ^b AB^	7.25 (±0.25) ^a‐c A^
E_6_	4.25 (±0.39) ^b C^	5.44 (±0.09) ^b B^	6.48 (±0.37) ^ab AB^	7.42 (±0.47) ^a‐c A^	4.05 (±0.26) ^bc C^	5.22 (±0.48) ^bc B^	6.25 (±0.29) ^b AB^	7.28 (±0.17) ^a‐c A^
E_7_	4.32 (±0.42) ^b B^	5.49 (±0.18) ^b B^	6.00 (±0.59) ^b B^	7.97 (±0.83) ^a‐c A^	4.02 (±0.07) ^bc C^	5.24 (±0.11) ^bc B^	5.75 (±0.60) ^bc B^	7.83 (±0.45) ^a‐c A^
E_8_	4.84 (±0.48) ^b B^	5.63 (±0.13) ^b B^	6.49 (±0.52) ^ab B^	8.37 (±0.87) ^a‐c A^	4.62 (±0.21) ^b D^	5.41 (±0.12) ^bc C^	6.47 (±0.26) ^b B^	8.11 (±0.23) ^a‐c A^
E_9_	4.33 (±0.27) ^b C^	5.32 (±0.34) ^bc C^	6.63 (±0.61) ^ab B^	8.65 (±0.50) ^ab A^	4.01 (±0.19) ^bc C^	5.06 (±0.35) ^bc C^	6.31 (±0.47) ^b B^	8.44 (±0.44) ^ab A^
E_10_	4.40 (±0.30) ^b C^	5.25 (±0.09) ^bc C^	6.55 (±0.64) ^ab B^	8.85 (±0.31) ^a A^	4.20 (±0.36) ^bc C^	5.21 (±0.30) ^bc BC^	6.29 (±0.36) ^b B^	8.63 (±0.80) ^ab A^
E_11_	4.48 (±0.28) ^b C^	5.21 (±0.18) ^bc BC^	6.50 (±0.49) ^ab B^	8.26 (±0.57) ^a‐c A^	4.23 (±0.42) ^bc C^	5.11 (±0.13) ^bc B^	5.74 (±0.18) ^bc B^	8.01 (±0.18) ^a‐c A^
E_12_	4.74 (±0.30) ^b C^	5.26 (±0.27) ^bc C^	6.59 (±0.53) ^ab B^	8.62 (±0.35) ^ab A^	4.42 (±0.28) ^bc C^	4.98 (±0.09) ^bc BC^	6.04 (±0.34) ^bc B^	8.50 (±0.69) ^ab A^
E_13_	5.01 (±0.32) ^b B^	5.26 (±0.30) ^bc B^	6.36 (±0.40) ^ab B^	8.75 (±0.66) ^ab A^	4.80 (±0.08) ^b C^	5.25 (±0.24) ^bc BC^	5.72 (±0.23) ^bc B^	8.44 (±0.20) ^ab A^
E_14_	4.57 (±0.40) ^b B^	5.29 (±0.21) ^bc B^	5.57 (±0.14) ^bc B^	8.15 (±0.42) ^a‐c A^	4.42 (±0.20) ^bc B^	5.27 (±0.27) ^bc B^	5.47 (±0.35) ^bc B^	7.84 (±0.72) ^a‐c A^
E_15_	4.85 (±0.50) ^b B^	5.28 (±0.16) ^bc B^	6.08 (±0.35) ^ab B^	8.65 (±0.55) ^ab A^	4.67 (±0.46) ^b C^	5.76 (±0.12) ^b B^	5.99 (±0.14) ^bc B^	8.55 (±0.44) ^ab A^
E_16_	3.86 (±0.29) ^b C^	4.73 (±0.17) ^c BC^	4.55 (±0.06) ^c B^	6.85 (±0.28) ^c A^	3.56 (±0.19) ^c C^	4.63 (±0.32) ^c B^	4.96 (±0.16) ^c B^	6.70 (±0.43) ^c A^
E_17_	4.68 (±0.38) ^b C^	5.50 (±0.25) ^b BC^	6.01 (±0.34) ^b B^	8.40 (±0.19) ^a‐c A^	4.59 (±0.19) ^b B^	5.36 (±0.23) ^bc B^	5.59 (±0.16) ^bc B^	8.32 (±0.67) ^a‐c A^
E_18_	6.31 (±0.05) ^a C^	6.28 (±0.10) ^a C^	7.43 (±0.30) ^a B^	8.96 (±0.36) ^a A^	6.19 (±0.26) ^a B^	6.59 (±0.12) ^a B^	7.53 (±0.45) ^a AB^	8.80 (±0.91) ^a A^
Species								
*longifolia*	4.51 (±0.11) ^bc D^	5.45 (±0.06) ^b C^	6.29 (±0.14) ^b B^	7.70 (±0.19) ^ab A^	4.28 (±0.10) ^b D^	5.22 (±0.10) ^bc C^	6.20 (±0.12) ^b B^	7.44 (±0.14) ^bc A^
*pulegium*	4.66 (±0.15) ^b D^	5.25 (±0.10) ^b C^	6.50 (±0.24) ^b B^	8.62 (±0.23) ^ab A^	4.41 (±0.15) ^b D^	5.14 (±0.10) ^bc C^	5.95 (±0.14) ^b B^	8.40 (±0.25) ^ab A^
*spicata*	4.71 (±0.30) ^b C^	5.28 (±0.12) ^b BC^	5.83 (±0.20) ^b B^	8.40 (±0.34) ^ab A^	4.54 (±0.24) ^b C^	5.51 (±0.17) ^b B^	5.73 (±0.20) ^b B^	8.19 (±0.42) ^ab A^
*rotundifolia*	3.86 (±0.29) ^c C^	4.73 (±0.17) ^c B^	4.55 (±0.06) ^c B^	6.85 (±0.28) ^b A^	3.56 (±0.19) ^c C^	4.63 (±0.32) ^c B^	4.96 (±0.16) ^c B^	6.70 (±0.43) ^c A^
*mozafariani*	4.68 (±0.38) ^b C^	5.50 (±0.25) ^b BC^	6.01 (±0.34) ^b B^	8.40 (±0.19) ^a A^	4.59 (±0.19) ^b B^	5.36 (±0.23) ^b B^	5.59 (±0.16) ^bc B^	8.32 (±0.67) ^ab A^
*piperita*	6.31 (±0.05) ^a C^	6.28 (±0.10) ^a C^	7.43 (±0.30) ^a B^	8.96 (±0.36) ^a A^	6.19 (±0.26) ^a B^	6.59 (±0.12) ^a B^	7.53 (±0.45) ^a AB^	8.80 (±0.91) ^a A^
**Total mean**	**4.64 (±0.09) ^A^**	**5.39 (±0.05) ^A^**	**6.24 (±0.11) ^A^**	**8.04 (±0.13) ^A^**	**4.42 (±0.09) ^B^**	**5.29 (±0.07) ^A^**	**6.06 (±0.09) ^A^**	**7.82 (±0.13) ^A^**

Note

Results are represented as mean ± standard error. Means in a column and row followed by the different superscripts are significantly different at *p* ≤ .05 using Tukey's test. Different superscript uppercase letters (within each row) show differences between the salinity stress levels within the same analysis group (*p* < .05). Different superscript lowercase letters (within each column) show differences between ecotypes within the same analysis day (*p* < .05).

**TABLE 10:** Antioxidant activity (%) related to different mint ecotypes and salinity stress levels at first and second harvest

Ecotypes	First harvest				Second harvest			
	Salt stress (dS/m)				Salt stress (dS/m)			
	0	2.5	5	7.5	0	2.5	5	7.5
E_1_	17.12 (±0.58) ^b‐d D^	29.57 (±0.70) ^b C^	42.31 (±0.58) ^b B^	63.25 (±0.58) ^b A^	17.40 (±0.58) ^c‐g D^	30.71 (±0.58) ^b C^	42.59 (±0.58) ^bc B^	63.53 (±0.58) ^bc A^
E_2_	17.88 (±0.83) ^b‐d D^	30.61 (±0.38) ^b C^	43.68 (±4.03) ^b B^	63.10 (±2.12) ^b A^	17.97 (±0.41) ^c‐g D^	30.71 (±0.53) ^b C^	42.78 (±0.32) ^bc B^	65.52 (±1.64) ^b A^
E_3_	16.32 (±0.28) ^b‐d D^	29.81 (±1.02) ^b C^	42.65 (±3.94) ^b B^	62.62 (±2.17) ^b A^	16.39 (±0.28) ^f‐h D^	30.34 (±1.05) ^b C^	45.48 (±1.31) ^b B^	62.50 (±0.87) ^bc A^
E_4_	18.25 (±0.42) ^a‐d D^	31.16 (±0.95) ^b C^	43.58 (±1.26) ^b B^	61.40 (±2.48) ^b A^	18.30 (±0.32) ^c‐f D^	30.71 (±1.06) ^b C^	43.32 (±1.24) ^b B^	61.00 (±1.63) ^bc A^
E_5_	17.65 (±0.71) ^b‐d D^	31.15 (±1.08) ^b C^	41.36 (±3.10) ^b B^	62.50 (±1.08) ^b A^	17.69 (±0.31) ^c‐g D^	31.42 (±0.73) ^b C^	41.50 (±1.44) ^bc B^	62.60 (±1.81) ^bc A^
E_6_	16.98 (±1.18) ^b‐d D^	30.28 (±1.57) ^b C^	41.85 (±3.87) ^b B^	63.85 (±2.21) ^b A^	17.22 (±0.40) ^d‐g D^	30.31 (±0.87) ^b C^	41.93 (±0.97) ^bc B^	64.04 (±1.11) ^bc A^
E_7_	17.52 (±1.72) ^b‐d D^	29.42 (±1.02) ^b C^	41.36 (±1.67) ^b B^	62.76 (±2.17) ^b A^	17.84 (±0.31) ^c‐g D^	29.71 (±0.51) ^b C^	41.40 (±0.72) ^bc B^	62.84 (±6.53) ^bc A^
E_8_	17.24 (±0.70) ^b‐d D^	30.65 (±2.65) ^b C^	41.74 (±0.72) ^b B^	62.65 (±1.81) ^b A^	17.40 (±0.70) ^c‐g D^	30.89 (±0.89) ^b C^	41.82 (±3.14) ^bc B^	62.70 (±5.79) ^bc A^
E_9_	16.85 (±1.36) ^b‐d D^	30.35 (±0.38) ^b C^	42.98 (±1.24) ^b B^	63.65 (±1.10) ^b A^	16.88 (±0.49) ^e‐g D^	30.70 (±0.89) ^b C^	43.15 (±2.74) ^b B^	63.75 (±1.84) ^bc A^
E_10_	15.95 (±1.29) ^cd D^	30.36 (±0.78) ^b C^	44.14 (±2.80) ^b B^	63.48 (±1.83) ^b A^	16.09 (±0.74) ^gh D^	30.39 (±0.53) ^b C^	44.34 (±3.07) ^b B^	63.78 (±6.26) ^bc A^
E_11_	17.41 (±0.30) ^b‐d D^	30.31 (±1.75) ^b C^	43.65 (±4.28) ^b B^	63.59 (±2.20) ^b A^	17.66 (±0.71) ^c‐g D^	30.55 (±1.06) ^b C^	43.81 (±2.28) ^b B^	63.78 (±5.52) ^bc A^
E_12_	18.96 (±1.75) ^a‐c D^	30.46 (±0.88) ^b C^	41.72 (±1.22) ^b B^	62.36 (±3.24) ^b A^	19.04 (±0.88) ^b‐d D^	30.79 (±1.24) ^b C^	42.06 (±0.73) ^bc B^	62.39 (±3.96) ^bc A^
E_13_	19.23 (±1.89) ^a‐c C^	31.96 (±1.85) ^ab B^	39.47 (±2.57) ^b B^	62.46 (±3.25) ^b A^	19.33 (±0.45) ^bc D^	30.76 (±0.53) ^b C^	42.50 (±1.96) ^bc B^	62.60 (±4.34) ^bc A^
E_14_	18.24 (±0.42) ^a‐d D^	30.76 (±0.27) ^b C^	40.91 (±0.87) ^b B^	63.15 (±2.55) ^b A^	18.55 (±0.43) ^b‐e D^	30.63 (±0.28) ^b C^	43.27 (±1.50) ^b B^	63.24 (±2.19) ^bc A^
E_15_	17.65 (±0.92) ^b‐d D^	31.41 (±0.54) ^b C^	41.65 (±0.96) ^b B^	63.62 (±2.20) ^b A^	17.87 (±0.52) ^c‐g D^	32.31 (±0.93) ^b C^	41.68 (±1.44) ^bc B^	60.71 (±0.81) ^bc A^
E_16_	14.71 (±0.51) ^d D^	24.65 (±0.62) ^c C^	36.97 (±2.08) ^b B^	58.07 (±1.42) ^b A^	14.81 (±0.68) ^h D^	29.59 (±1.02) ^b C^	37.23 (±1.16) ^c B^	53.17 (±1.85) ^c A^
E_17_	20.00 (±2.08) ^ab D^	30.71 (±0.53) ^b C^	42.32 (±2.44) ^b B^	62.35 (±2.52) ^b A^	20.25 (±0.47) ^ab D^	31.04 (±0.54) ^b C^	42.62 (±1.48) ^bc B^	61.03 (±2.53) ^bc A^
E_18_	21.56 (±0.51) ^a D^	34.82 (±0.58) ^a C^	56.93 (±1.46) ^a B^	73.18 (±0.61) ^a A^	21.86 (±1.14) ^a D^	35.09 (±0.45) ^a C^	53.86 (±1.19) ^a B^	80.07 (±2.88) ^a A^
Species								
*longifolia*	17.31 (±0.30) ^c D^	30.33 (±0.38) ^b C^	42.39 (±0.79) ^b B^	62.86 (±0.56) ^b A^	17.45 (±0.16) ^c D^	30.61 (±0.25) ^b C^	42.66 (±0.52) ^b B^	63.16 (±0.95) ^b A^
*pulegium*	17.89 (±0.73) ^bc D^	30.77 (±0.65) ^b C^	42.25 (±1.39) ^b B^	62.97 (±1.22) ^b A^	18.03 (±0.46) ^c D^	30.62 (±0.40) ^b C^	43.18 (±1.00) ^b B^	63.14 (±2.29) ^b A^
*spicata*	17.94 (±0.48) ^bc D^	31.08 (±0.30) ^b C^	41.28 (±0.62) ^b B^	63.39 (±1.56) ^b A^	18.21 (±0.34) ^c D^	31.47 (±0.55) ^b C^	42.48 (±1.01) ^b B^	61.97 (±1.18) ^b A^
*rotundifolia*	14.71 (±0.51) ^d D^	24.65 (±0.62) ^c C^	36.97 (±2.08) ^b B^	58.07 (±1.42) ^c A^	14.81 (±0.68) ^d D^	29.59 (±1.02) ^b C^	37.23 (±1.16) ^c B^	53.17 (±1.85) ^c A^
*mozafariani*	20.00 (±2.08) ^ab D^	30.71 (±0.53) ^b C^	42.32 (±2.44) ^b B^	62.35 (±2.52) ^bc A^	20.25 (±0.47) ^b D^	31.04 (±0.54) ^b C^	42.62 (±1.48) ^b B^	61.03 (±2.53) ^b A^
*piperita*	21.56 (±0.51) ^a D^	34.82 (±0.58) ^a C^	56.93 (±1.46) ^a B^	73.18 (±0.61) ^a A^	21.86 (±1.14) ^a D^	35.09 (±0.45) ^a C^	53.86 (±1.19) ^a B^	80.07 (±2.88) ^a A^
**Total mean**	**17.75 (±0.29) ^A^**	**30.47 (±0.32) ^A^**	**42.74 (±0.68) ^A^**	**63.22 (±0.54) ^A^**	**17.92 (±0.22) ^A^**	**30.92 (±0.22) ^A^**	**43.07 (±0.51) ^A^**	**63.29 (±0.91) ^A^**

Note

Results are represented as mean ± standard error. Means in a column and row followed by the different superscripts are significantly different at *p* ≤ .05 using Tukey's test. Different superscript uppercase letters (within each row) show differences between the salinity stress levels within the same analysis group (*p* < .05). Different superscript lowercase letters (within each column) show differences between ecotypes within the same analysis day (*p* < .05).

The highest contents of total phenols and flavonoids in both harvest stages were found in E18 ecotype, while the lowest contents were related to E16 (Tables 8 and 9). In addition, the total phenolic and flavonoid contents were affected by the salinity stress in both harvest stages. It was also observed that with increasing the salinity stress, the total phenolic and flavonoid contents increased in all ecotypes.

The highest levels of antioxidant activity among the ecotypes at all stress levels, in both harvest stages, were found in E18 ecotype (Table 10). In contrast, the lowest antioxidant activity in the first and second harvest stages at the control and 2.5 dS/m levels was related to E16 ecotype. The statistical analysis revealed that there was no significant difference in the amount of antioxidant activity at all levels of salinity stress between the harvest stages. However, with increasing the salinity stress, the antioxidant activity increased in all ecotypes in both harvest stages.

Mint is a rich source of phenols and flavonoids, which are very important (Khalvandi et al., 2019; Riachi & De Maria, 2015). The results of the present experiment showed that the amount of these compounds increased significantly in salinity stress conditions in comparison with the control treatment.

Total phenolic and total flavonoid content usually act as secondary antioxidants and free radical scavengers under the salinity stress (Khalvandi et al., 2019). Several studies have reported that the mint has a high total phenolic and flavonoid content compound (Riachi & De Maria, 2015) and therefore exhibits good antioxidant activity (Khalvandi et al., 2019). There is a strong relationship between the amount of total phenolic and flavonoid content in plant tissue and the intensity of stress to which plants are exposed (Waśkiewicz et al., 2013). In fact, the plant tolerance is enhanced by total phenolic and total flavonoid content compounds as well as antioxidants. An increase in the synthesis of phenolic compounds under the influence of salinity stress has been reported in various plants of the mint family (Khalvandi et al., 2019; Taïbi et al., 2016; Yuan et al., 2010).

These compounds are strong antioxidants in plant tissues under stress, and this property is due to the chemical structure and phenolic group of these metabolites, thus reducing oxidative damage and protecting cellular structures from the negative effects of salinity stress (Boscaiu et al., 2010; Waśkiewicz et al., 2013).

In this study, the content of total phenolic compounds increased with salinity stress in different mint species. It was also observed that the content of total phenolic compounds is higher in salt‐tolerant species. Previous studies demonstrated that high total phenolic content is one of the characteristics of salt‐tolerant ecotypes (Falleh et al., 2012; Khalvandi et al., 2019). The whole leaf and especially root total phenolic content usually increases in the tolerant species or cultivars as a defense mechanism to reduce the sodium ions and active oxygen species (Al Kharusi et al., 2019). Salinity stress generates a lot of excited energy, and one way to dissipate this energy is to produce polyphenols, especially in photosynthetic structures (Waśkiewicz et al., 2013). Increased biosynthesis of flavonoids is also associated with an increase in glutathione s‐transferase enzyme, which is involved in the transfer of flavonoids to the vacuole to inhibit active oxygen species (Czerniawski & Bednarek, 2018). The results also revealed that the salinity stress may increase antioxidant activity in mint. Khalvandi et al. (2019) showed that the salinity stress increased DPPH‐scavenging activity in peppermint. Our results showed a strong relationship between total phenolic and total flavonoid contents and antioxidant activity in mint. This direct relationship is more important at higher salinity stress levels. In salt‐tolerant ecotypes, higher levels of antioxidant activity were observed, indicating the effect of this nonenzymatic antioxidant in salinity stress conditions. It has also been reported that at high stress levels, the antioxidant activity plays a major role in the resistance to salinity stress in salt‐tolerant ecotypes (Khalvandi et al., 2019; Valifard et al., 2014), which is consistent with the results of the present study. Some studies have shown that antioxidant activity plays an important role in salinity stress resistance by trapping and inhibiting free radicals (e.g., Kaur et al., 2014).

### 3.3 Dry matter

The results of the present experiment showed that there was a significant difference in the amount of dry matter between the different species at all levels of the salinity stress (Table 11). Evaluation of dry matter production at different levels of salinity stress showed that the amount of dry matter in *piperita* was not affected by the salinity stress. The amount of dry matter produced in other species decreased with increasing the salinity stress. Also, the amount of dry matter in the second harvest was higher than the first harvest at all levels of salinity stress.

**TABLE 11:** Dry matter weight (g) related to different mint ecotypes and salinity stress levels at first and second harvest

Ecotypes	First harvest				Second harvest			
	Salt stress (dS/m)				Salt stress (dS/m)			
	0	2.5	5	7.5	0	2.5	5	7.5
E_1_	9.36 (±0.40) ^d A^	6.83 (±0.33) ^e B^	6.08 (±0.29) ^e B^	4.40 (±0.27) ^e C^	9.57 (±0.51) ^e A^	6.97 (±0.30) ^e B^	6.15 (±0.29) ^e B^	4.44 (±0.58) ^cd C^
E_2_	5.97 (±0.06) ^hi A^	4.30 (±0.04) ^j B^	3.82 (±0.01) ^jk C^	2.86 (±0.06) ^j D^	6.12 (±0.13) ^jk A^	4.34 (±0.08) ^jk B^	3.87 (±0.06) ^ij C^	2.89 (±0.07) ^h‐j D^
E_3_	6.23 (±0.09) ^h A^	4.36 (±0.09) ^j B^	4.05 (±0.02) ^ij C^	2.86 (±0.07) ^j D^	6.38 (±0.09) ^ij A^	4.41 (±0.04) ^jk B^	4.10 (±0.04) ^hi C^	2.88 (±0.08) ^h‐j D^
E_4_	5.40 (±0.06) ^j A^	4.05 (±0.11) ^j B^	3.62 (±0.05) ^kl C^	2.65 (±0.07) ^j D^	5.51 (±0.05) ^l A^	4.10 (±0.05) ^k B^	3.66 (±0.06) ^jk C^	2.66 (±0.08) ^j D^
E_5_	8.30 (±0.09) ^e A^	6.31 (±0.12) ^fg B^	5.23 (±0.02) ^fg C^	4.07 (±0.07) ^fg D^	8.50 (±0.13) ^f A^	6.41 (±0.08) ^fg B^	5.27 (±0.05) ^fg C^	4.10 (±0.09) ^d‐f D^
E_6_	5.64 (±0.08) ^ij A^	4.40 (±0.05) ^j AB^	3.50 (±0.03) ^l C^	2.71 (±0.01) ^j D^	5.77 (±0.13) ^kl A^	4.49 (±0.03) ^j B^	3.53 (±0.01) ^k C^	2.74 (±0.05) ^ij D^
E_7_	6.77 (±0.12) ^g A^	5.01 (±0.06) ^i B^	4.13 (±0.02) ^i C^	3.25 (±0.06) ^i D^	6.92 (±0.07) ^hi A^	5.11 (±0.04) ^i B^	4.20 (±0.03) ^h C^	3.28 (±0.06) ^gh D^
E_8_	9.11 (±0.12) ^d A^	6.56 (±0.06) ^ef B^	6.10 (±0.02) ^e C^	4.46 (±0.06) ^e D^	9.32 (±0.29) ^e A^	6.69 (±0.06) ^ef B^	6.18 (±0.09) ^e C^	4.52 (±0.08) ^cd D^
E_9_	6.82 (±0.26) ^g A^	5.18 (±0.05) ^i B^	4.23 (±0.02) ^i C^	3.20 (±0.07) ^i D^	6.96 (±0.19) ^hi A^	5.28 (±0.06) ^i B^	4.28 (±0.06) ^h C^	3.24 (±0.08) ^g‐i D^
E_10_	7.70 (±0.08) ^f A^	6.31 (±0.04) ^fg B^	5.39 (±0.07) ^f C^	3.85 (±0.04) ^gh D^	7.85 (±0.17) ^g A^	6.39 (±0.11) ^fg B^	5.47 (±0.04) ^f C^	3.90 (±0.11) ^ef D^
E_11_	7.07 (±0.12) ^g A^	5.87 (±0.15) ^h B^	4.88 (±0.03) ^h C^	3.60 (±0.06) ^h D^	7.21 (±0.15) ^h A^	5.94 (±0.09) ^h B^	4.96 (±0.12) ^g C^	3.65 (±0.11) ^fg D^
E_12_	7.28 (±0.19) ^fg A^	5.90 (±0.04) ^h B^	5.02 (±0.01) ^gh C^	3.71 (±0.04) ^h D^	7.42 (±0.15) ^gh A^	5.98 (±0.09) ^h B^	5.09 (±0.10) ^g C^	3.77 (±0.07) ^fg D^
E_13_	11.50 (±0.20) ^b A^	9.20 (±0.07) ^b B^	7.82 (±0.02) ^b C^	5.75 (±0.05) ^b D^	11.80 (±0.14) ^bc A^	9.35 (±0.07) ^b B^	7.92 (±0.08) ^b C^	5.80 (±0.13) ^b D^
E_14_	10.42 (±0.19) ^c A^	8.23 (±0.08) ^d B^	6.77 (±0.07) ^d C^	4.90 (±0.09) ^d D^	10.69 (±0.19) ^d A^	8.34 (±0.08) ^d B^	6.88 (±0.07) ^d C^	4.94 (±0.11) ^c D^
E_15_	11.33 (±0.20) ^b A^	8.83 (±0.14) ^c B^	7.14 (±0.03) ^c C^	5.55 (±0.11) ^bc D^	11.61 (±0.21) ^c A^	8.97 (±0.09) ^c B^	7.24 (±0.13) ^c C^	5.60 (±0.13) ^b D^
E_16_	12.03 (±0.35) ^a A^	8.42 (±0.10) ^d B^	7.34 (±0.10) ^c C^	5.41 (±0.08) ^c D^	12.31 (±0.13) ^ab A^	8.56 (±0.09) ^d B^	7.44 (±0.10) ^c C^	5.46 (±0.16) ^b D^
E_17_	8.25 (±0.11) ^e A^	6.02 (±0.13) ^gh B^	4.95 (±0.05) ^h C^	4.29 (±0.14) ^ef D^	8.42 (±0.07) ^f A^	6.14 (±0.08) ^gh B^	5.01 (±0.04) ^g C^	4.32 (±0.12) ^de D^
E_18_	12.26 (±0.04) ^a A^	10.42 (±0.11) ^a B^	8.95 (±0.08) ^a C^	7.11 (±0.16) ^a D^	12.50 (±0.26) ^a A^	10.63 (±0.20) ^a B^	9.05 (±0.19) ^a C^	7.16 (±0.21) ^a D^
Species								
*longifolia*	7.06 (±0.24) ^b A^	5.22 (±0.18) ^d B^	4.53 (±0.16) ^d C^	3.38 (±0.12) ^d D^	7.23 (±0.25) ^b A^	5.31 (±0.18) ^d B^	4.58 (±0.17) ^d C^	3.42 (±0.13) ^d D^
*pulegium*	8.39 (±0.47) ^b A^	6.82 (±0.36) ^c B^	5.78 (±0.31) ^c C^	4.23 (±0.23) ^c D^	8.57 (±0.49) ^b A^	6.91 (±0.37) ^c B^	5.86 (±0.31) ^c C^	4.28 (±0.23) ^c D^
*spicata*	10.87 (±0.21) ^a A^	8.53 (±0.14) ^b B^	6.95 (±0.08) ^b C^	5.22 (±0.14) ^b D^	11.15 (±0.22) ^a A^	8.65 (±0.13) ^b B^	7.06 (±0.10) ^b C^	5.27 (±0.15) ^b D^
*rotundifolia*	12.03 (±0.35) ^a A^	8.42 (±0.10) ^b B^	7.34 (±0.10) ^b C^	5.41 (±0.08) ^b D^	12.31 (±0.13) ^a A^	8.56 (±0.09) ^b B^	7.44 (±0.10) ^b C^	5.46 (±0.16) ^b D^
*mozafariani*	8.25 (±0.11) ^b D^	6.02 (±0.13) ^cd C^	4.95 (±0.05) ^cd B^	4.29 (±0.14) ^c A^	8.42 (±0.07) ^b A^	6.14 (±0.08) ^cd B^	5.01 (±0.04) ^cd C^	4.32 (±0.12) ^c D^
*piperita*	12.26 (±0.04) ^a A^	10.42 (±0.11) ^a B^	8.95 (±0.08) ^a C^	7.11 (±0.16) ^a D^	12.50 (±0.26) ^a D^	10.63 (±0.20) ^a C^	9.05 (±0.19) ^a B^	7.16 (±0.21) ^a A^
**Total mean**	**8.41 (±0.26) ^B^**	**6.45 (±0.22) ^B^**	**5.50 (±0.18) ^B^**	**4.15 (±0.14) ^A^**	**8.60 (±0.27) ^A^**	**6.56 (±0.22) ^A^**	**5.57 (±0.19) ^A^**	**4.19 (±0.15) ^A^**

Note

Results are represented as mean ± standard error. Means in a column and row followed by the different superscripts are significantly different at *p* ≤ .05 using Tukey's test. Different superscript uppercase letters (within each row) show differences between the salinity stress levels within the same analysis group (*p* < .05). Different superscript lowercase letters (within each column) show differences between ecotypes within the same analysis day (*p* < .05).

The highest amount of dry matter produced by E18 in both harvest stages in all salinity stress levels. In the first harvest, the lowest amount of dry matter at the control levels and 7.5 dS/m was observed in E4. In addition, in the first harvest stage at 2.5 and 5 dS/m salinity stress levels, the lowest dry matter amount was found in E2 and E6, respectively. At the second harvest stage in control, 2.5 and 7.5 dS/m levels, the lowest amount of dry matter was observed in E4. At the second harvest, the lowest amount of dry matter produced at the 5 dS/m salinity stress level was observed in E6. Comparison of the main effect of harvest stages showed that the dry matter produced at the control, 2.5 and 5 dS/m salinity stress levels was affected by the harvest stage.

In addition, in the second harvest, the plant had its rootstock; and it had made enough branches and spent more energy to produce the dry matter. Growth and yield are strongly influenced by the genotype and environmental factors. The amount of the produced dry matter was affected by salinity stress; with increasing the stress level, the amount of dry matter produced in all ecotypes was reduced in both harvest stages. The amount of dry matter production is directly related to the yield of the photosynthetic pigments (Cui et al., 2016). As the yield of the photosynthetic pigments deteriorates under salinity stress, it reduces the production of the plant's dry matter. Also, under salinity stress, the leaf area of plants decreases, which also plays a significant role in reduction of dry matter production (Bacha et al., 2017). Absorption of nutrients and water is also impaired under salinity stress conditions, which has a very important effect on reducing dry matter yield (Parvaiz & Satyawati, 2008). Another factor affecting the reduction in dry matter production is the waste of plant energy to deal with adverse conditions during the exposure to the salinity stress (Parihar et al., 2015). Several studies have shown that under the salinity stress, the amount of the produced dry matter decreases, which is consistent with the results of the present experiment (Jahanet al., 2020; Pessarakli et al., 1989; Torabian et al., 2016).

### 3.4 Essential oil content

The highest and lowest essential oil content in all salinity stress levels were found in *piperita* and *rotundifolia* species, respectively (Table 12). Comparison of the two harvests showed that in the second harvest, a higher essential oil content was obtained, which could be due to the harvest of plants in the flowering stage.

**TABLE 12:** Essential oil content (%) related to different mint ecotypes and salinity stress levels at first and second harvest

Ecotypes	First harvest				Second harvest			
	Salt stress (dS/m)				Salt stress (dS/m)			
	0	2.5	5	7.5	0	2.5	5	7.5
E_1_	0.47 (±0.10) ^a‐c D^	1.11 (±0.05) ^c‐f C^	1.79 (±0.13) ^b B^	2.84 (±0.25) ^ab A^	0.50 (±0.10) ^cd D^	1.17 (±0.22) ^c C^	2.13 (±0.16) ^b B^	2.93 (±0.26) ^ab A^
E_2_	0.60 (±0.06) ^a D^	1.45 (±0.04) ^a C^	1.90 (±0.06) ^ab B^	2.74 (±0.06) ^a‐d A^	0.65 (±0.02) ^ab D^	1.52 (±0.12) ^a C^	2.16 (±0.09) ^b B^	2.83 (±0.08) ^a‐e A^
E_3_	0.56 (±0.05) ^a‐c D^	1.10 (±0.04) ^c‐f C^	2.09 (±0.09) ^ab B^	2.44 (±0.04) ^d‐f A^	0.58 (±0.03) ^a‐d D^	1.12 (±0.06) ^c C^	2.14 (±0.07) ^b B^	2.51 (±0.12) ^e‐g A^
E_4_	0.47 (±0.03) ^a‐c D^	1.08 (±0.03) ^d‐f C^	1.87 (±0.08) ^ab B^	2.66 (±0.06) ^a‐e A^	0.53 (±0.03) ^b‐d D^	1.12 (±0.08) ^c C^	1.95 (±0.05) ^b B^	2.70 (±0.09) ^b‐f A^
E_5_	0.41 (±0.04) ^c D^	1.28 (±0.04) ^b C^	1.99 (±0.20) ^ab B^	2.78 (±0.06) ^a‐c A^	0.49 (±0.03) ^cd D^	1.34 (±0.06) ^a‐c C^	2.02 (±0.05) ^b B^	2.80 (±0.06) ^a‐e A^
E_6_	0.42 (±0.03) ^bc D^	1.12 (±0.03) ^c‐f C^	1.99 (±0.10) ^ab B^	2.45 (±0.07) ^d‐f A^	0.47 (±0.01) ^d D^	1.15 (±0.09) ^c C^	2.07 (±0.22) ^b B^	2.53 (±0.10) ^d‐g A^
E_7_	0.53 (±0.05) ^a‐c C^	1.04 (±0.04) ^ef B^	2.08 (±0.20) ^ab A^	2.43 (±0.08) ^d‐f A^	0.60 (±0.06) ^a‐d D^	1.06 (±0.07) ^c C^	2.05 (±0.08) ^b B^	2.51 (±0.07) ^e‐g A^
E_8_	0.45 (±0.04) ^a‐c D^	1.15 (±0.05) ^b‐e C^	2.03 (±0.08) ^ab B^	2.78 (±0.05) ^a‐c A^	0.52 (±0.04) ^b‐d D^	1.20 (±0.02) ^bc C^	2.11 (±0.15) ^b B^	2.85 (±0.07) ^a‐e A^
E_9_	0.56 (±0.05) ^a‐c D^	1.17 (±0.05) ^b‐e C^	1.92 (±0.17) ^ab B^	2.40 (±0.04) ^ef A^	0.57 (±0.02) ^a‐d D^	1.21 (±0.03) ^bc C^	1.96 (±0.16) ^b B^	2.43 (±0.04) ^fg A^
E_10_	0.59 (±0.05) ^a D^	1.27 (±0.04) ^b C^	2.07 (±0.11) ^ab B^	2.50 (±0.04) ^c‐e A^	0.63 (±0.05) ^a‐c D^	1.28 (±0.03) ^a‐c C^	2.14 (±0.09) ^b B^	2.53 (±0.07) ^d‐g A^
E_11_	0.58 (±0.04) ^ab D^	1.23 (±0.05) ^bc C^	1.92 (±0.11) ^ab B^	2.86 (±0.12) ^a A^	0.64 (±0.07) ^ab D^	1.26 (±0.09) ^a‐c C^	2.01 (±0.15) ^b B^	2.91 (±0.15) ^a‐c A^
E_12_	0.48 (±0.03) ^a‐c D^	1.21 (±0.06) ^b‐d C^	2.07 (±0.05) ^ab B^	2.49 (±0.04) ^c‐f A^	0.55 (±0.05) ^b‐d D^	1.29 (±0.05) ^a‐c C^	2.14 (±0.04) ^b B^	2.52 (±0.07) ^e‐g A^
E_13_	0.57 (±0.06) ^a‐c D^	1.09 (±0.02) ^d‐f C^	1.99 (±0.18) ^ab B^	2.79 (±0.06) ^a‐c A^	0.64 (±0.01) ^ab D^	1.12 (±0.08) ^c C^	2.02 (±0.16) ^b B^	2.87 (±0.08) ^a‐d A^
E_14_	0.59 (±0.04) ^a D^	1.44 (±0.03) ^a C^	2.05 (±0.13) ^ab B^	2.87 (±0.13) ^a A^	0.63 (±0.03) ^a‐c D^	1.48 (±0.10) ^ab C^	2.11 (±0.17) ^b B^	2.88 (±0.08) ^a‐c A^
E_15_	0.45 (±0.03) ^a‐c D^	1.18 (±0.03) ^b‐d C^	1.97 (±0.14) ^ab B^	2.54 (±0.07) ^b‐e A^	0.52 (±0.04) ^b‐d D^	1.19 (±0.10) ^bc C^	2.04 (±0.05) ^b B^	2.57 (±0.07) ^c‐f A^
E_16_	0.41 (±0.04) ^c D^	1.01 (±0.04) ^f C^	1.42 (±0.11) ^c B^	2.19 (±0.05) ^f A^	0.46 (±0.03) ^d D^	1.09 (±0.09) ^c C^	1.59 (±0.06) ^c B^	2.21 (±0.06) ^g A^
E_17_	0.46 (±0.04) ^a‐c D^	1.27 (±0.04) ^b C^	1.97 (±0.16) ^ab B^	2.68 (±0.05) ^a‐e A^	0.55 (±0.02) ^b‐d D^	1.28 (±0.07) ^a‐c C^	1.95 (±0.04) ^b B^	2.76 (±0.08) ^a‐f A^
E_18_	0.61 (±0.06) ^a D^	1.48 (±0.04) ^a C^	2.28 (±0.09) ^a B^	2.94 (±0.14) ^a A^	0.70 (±0.01) ^a D^	1.54 (±0.03) ^a C^	2.51 (±0.06) ^a B^	3.08 (±0.07) ^a A^
Species								
*longifolia*	0.50 (±0.02) ^a‐c D^	1.17 (±0.02) ^b C^	1.96 (±0.04) ^b B^	2.61 (±0.04) ^b A^	0.55 (±0.02) ^bc D^	1.21 (±0.04) ^b C^	2.07 (±0.04) ^b B^	2.68 (±0.04) ^b A^
*pulegium*	0.56 (±0.02) ^ab D^	1.20 (±0.03) ^b C^	2.01 (±0.06) ^ab B^	2.66 (±0.05) ^ab A^	0.62 (±0.02) ^ab D^	1.24 (±0.04) ^b C^	2.08 (±0.06) ^b B^	2.70 (±0.07) ^b A^
*spicata*	0.52 (±0.03) ^a‐c D^	1.31 (±0.05) ^b C^	2.01 (±0.09) ^ab B^	2.71 (±0.09) ^ab A^	0.57 (±0.03) ^b D^	1.33 (±0.08) ^ab C^	2.07 (±0.08) ^b B^	2.73 (±0.08) ^b A^
*rotundifolia*	0.41 (±0.04) ^c D^	1.01 (±0.04) ^c C^	1.42 (±0.11) ^c B^	2.19 (±0.05) ^c A^	0.46 (±0.03) ^c D^	1.09 (±0.09) ^b C^	1.59 (±0.06) ^c B^	2.21 (±0.06) ^c A^
*mozafariani*	0.46 (±0.04) ^bc D^	1.27 (±0.04) ^b C^	1.97 (±0.16) ^b B^	2.68 (±0.05) ^ab A^	0.55 (±0.02) ^bc D^	1.28 (±0.07) ^b C^	1.95 (±0.04) ^b B^	2.76 (±0.08) ^b A^
*piperita*	0.61 (±0.06) ^a D^	1.48 (±0.04) ^a C^	2.28 (±0.09) ^a B^	2.94 (±0.14) ^a A^	0.70 (±0.01) ^a D^	1.54 (±0.03) ^a C^	2.51 (±0.06) ^a B^	3.08 (±0.07) ^a A^
**Total mean**	**0.51 (±0.01) ^B^**	**1.20 (±0.02) ^A^**	**1.97 (±0.03) ^B^**	**2.63 (±0.03) ^A^**	**0.57 (±0.01) ^A^**	**1.25 (±0.02) ^A^**	**2.06 (±0.03) ^A^**	**2.69 (±0.03) ^A^**

Note

Results are represented as mean ± standard error. Means in a column and row followed by the different superscripts are significantly different at *p* ≤ .05 using Tukey's test. Different superscript uppercase letters (within each row) show differences between the salinity stress levels within the same analysis group (*p* < .05). Different superscript lowercase letters (within each column) show differences between ecotypes within the same analysis day (*p* < .05).

In both harvest stages at all stress levels, the highest content of essential oil was observed in E18 while the lowest amount was found in E16 ecotype (Table 12). The essential oil content in the control treatment and 5 dS/m level was affected by the salinity stress. At the second harvest stage, the control treatment and 5 dS/m level had a higher essential oil content. The results revealed that at each harvest stages, the essential oil content increases with increasing the salinity stress. Ecological and genetic factors are among the most important variables that affect the growth, development, and biosynthesis of primary and secondary metabolites in medicinal plants (Li et al., 2020). Although these metabolites are essentially made by genetic processes, their production is also significantly influenced by environmental factors. The influence of environmental factors, such as biotic and abiotic stresses on essential oil content, depends on the type of essential oil storage organ and the chemical nature of the essential oil (Naik & Al‐Khayri, 2016). Under salinity stress conditions, the plant devotes energy to the biochemical settings required for survival instead of expending energy on growth and yield performance (Hancioglu et al., 2019).

In addition, in saline conditions, there is a strong correlation between root water and biomass production (Taarit et al., 2011). Thus, the reduction of water uptake by roots is associated with a decrease in the uptake of nutrients and their use by plants and can affect the production of dry matter (Razmjoo et al., 2008). Numerous studies on the mint have shown that the essential oil content increases under salinity stress conditions, which is consistent with the present experiment (Aziz et al., 2008; Kasrati et al., 2014; Khalvandi et al., 2019; Yu et al., 2015). The production of higher amounts of essential oils is one of the plant's solutions to increase the resistance against the salinity stress (Khalvandi et al., 2019). The results showed that in ecotypes where the highest essential oil content was found, the highest amount of dry matter, total phenolic content compounds, and antioxidant activity were also observed. In addition, photosynthetic pigments in ecotypes that have higher essential oil content were significantly higher comparing to the sensitive ecotypes. Since the production of secondary metabolites originates from sugars, the function of the photosynthetic system is of great importance in this pathway (Bourgaud et al., 2001; Fig ueiredo et al., 2008; Isah, 2019; Naik & Al‐Khayri, 2016; Verpoorte & Memelink, 2002).

### 3.5 Cluster and principal component analysis

**FIGURE 2:**
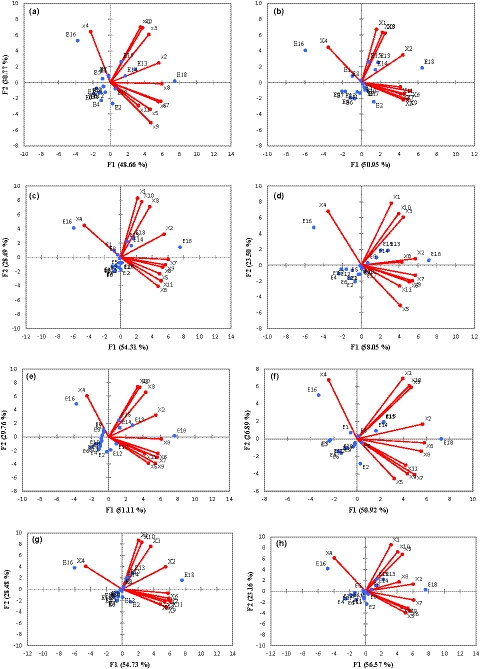
Biplots derived from principal component analysis based on first and second components. Biplots resulted from the all studied traits in 18 mint ecotypes. (A), control at first harvest; (B), 2.5 dS/m at first harvest; (C), 5 dS/m first harvest; (D), 7.5 dS/m first harvest; (E), control at second harvest; (F), 2.5 dS/m at second harvest; (G), 5 dS/m second harvest; (H), 7.5 dS/m second harvest. (X1), chlorophyll a; (X2), chlorophyll b; (X3), total chlorophyll; (X4), chlorophyll a/b ratio; (X5), carotenoid; (X6), antioxidant activity; (X7), total phenolic content; (X8), total flavonoid content; (X9), total anthocyanin; (X10), dry matter weight; (X11), essential oil content

The results of cluster analysis for different ecotypes of mint were shown in Figure 1 (separately by stress levels and first and second harvest stages). The results for the first harvest stage showed that the control, 2.5, 5, and 7.5 dS/m levels ecotypes were divided into 3, 4, 5, and 7 separate groups, respectively. Also, in the case of the second harvest stage, 3, 3, 5, and 5 groups were separated, respectively. The results also revealed that with increasing the salinity stress, the ecotypes were separated into further groups, indicating that the salinity stress caused more pronounced differences among the ecotypes. The first harvest stage had a higher variety at 2.5, 5, and 7.5 dS/m stress levels. In the first harvest stage, E18 and E16 were placed in an independent group by applying salinity stress of 2.5, 5, and 7.5 dS/m. But in the second harvest, only at 5 and 7.5 dS/m levels, the E18 and E16 ecotypes were grouped separately and individually. The results of cluster analysis showed that the salinity stress in the first harvest stage is capable of revealing the distinction between ecotypes. But in the second harvest stage, the difference between the ecotypes was not as obvious as in the first harvest stage. Principal component analysis showed that the relative variance of the first component in the first harvest stage was affected by the salinity stress, as with increasing the salinity stress, the ratio of variance in the first component increased (Figure 2). However, in the second harvest stage, there was no clear difference between the relative variance of the first component at the control treatment and the salinity stress of 2.5 dS/m. With increasing the salinity stress at 5 and 7.5 dS/m, the ratio of variance in the first component increased. Based on the results of the principal component analysis, it was observed that the E16 and E18 ecotypes are located separately from the other ecotypes. The biplot also showed an increasing distance between the two ecotypes. Ecotype E16 is closely associated with the chlorophyll a/b ratio variable. In the first harvest stage, E18 ecotype is strongly correlated with chlorophyll b, total phenolic content, and total anthocyanin, but in the second harvest stage, it was observed that E18 ecotype is strongly correlated with chlorophyll b, total phenolic content, antioxidant activity, and total anthocyanin which indicates the importance of this ecotype as the superior ecotype. In addition, it is observed that the grouping based on principal component analysis is to some extent consistent with the clustering results. Several studies have used principal component analysis and cluster analysis for grouping of ecotypes (Babaei & Ajdanian, 2020; Gaafar et al., 2017; Kumar et al., 2019).

## 4 CONCLUSION

Evaluation of different species under salinity stress conditions is a solution for selecting superior species in order to produce more dry matter and phytochemical compounds. The present experiment showed that there was a favorable diversity between ecotypes and species in terms of all the studied traits. It was also observed that the production of dry matter and essential oils had a direct relationship with the yield of the photosynthetic pigments. Salinity stress increased the levels of total anthocyanin, total phenolic and flavonoids content, antioxidant activity, and the essential oil content. Overall, the salt‐tolerant ecotypes and species had higher levels of photosynthetic pigments and secondary metabolites. The *piperita* species, as the superior species under salinity stress, had certain characteristics that have a strong relationship with photosynthetic pigments, total phenolic and flavonoids content, antioxidant activity, total anthocyanin, and essential oil content.

## CONFLICT OF INTERESTS

The authors declare that they have no competing interests.

## AUTHORS’ CONTRIBUTIONS

SJH, ZTS, and HP were involved in designed and conducted the research, data collection, analysis of results, and writing‐original draft manuscript. SJH, ZT, HP, AMB, SAMMS, SN, and SH were involved in development of the study design and data curation. SJH, SH, and AM were involved in development of the analysis of results and contributed to submitting the manuscript. SJH, ZT, AM, SAMMS, HP, SH, and SN were reviewed and revised the manuscript.

## Consent for publication

Not applicable.

## Data Availability

Research data are not shared.
